# Structural and Biological Insights Into Copper(I) Phosphane Complexes With Boron‐Phenylated Poly(3‐(CF_3_)Pyrazolyl)‐ and Poly(6‐(CF_3_)‐2‐pyridyl)‐Borates

**DOI:** 10.1002/chem.70848

**Published:** 2026-03-04

**Authors:** Vo Quang Huy Phan, Jo’ Del Gobbo, Mukundam Vanga, Deepika V. Karade, Cristina Marzano, Valentina Gandin, Laura Rigon, Carlo Santini, Maura Pellei, H. V. Rasika Dias

**Affiliations:** ^1^ Department of Chemistry and Biochemistry The University of Texas at Arlington Arlington Texas USA; ^2^ School of Science and Technology Chemistry Division University of Camerino Camerino Italy; ^3^ Department of Pharmaceutical and Pharmacological Sciences University of Padova Padova Italy; ^4^ Department of Medicine University of Padova Padova Italy

**Keywords:** copper(I), cytotoxicity, fluorinated chelate ligands, phosphanes, scorpionates, spectroscopy, X‐ray

## Abstract

Six new copper(I) phosphane complexes supported by *B*‐phenylated poly(3‐(CF_3_)pyrazolyl)‐ and poly(6‐(CF_3_)‐2‐pyridyl)borate ligands as [Ph_3_B(3‐(CF_3_)Pz)]^−^ (**1**), [Ph_2_B(3‐(CF_3_)Pz)_2_]^−^ (**2**), [PhB(3‐(CF_3_)Pz)_3_]^−^ (**3**), [Ph_3_B(6‐(CF_3_)Py)]^−^ (**4**), [Ph_2_B(6‐(CF_3_)Py)_2_]^−^ (**5**) and [PhB(6‐(CF_3_)Py)_3_]^−^ (**6**) have been synthesized to systematically modulate steric and electronic properties at copper(I), (Pz = pyrazolyl, Py = pyridyl). The complexes [Cu(PPh_3_){Ph_3_B(3‐(CF_3_)Pz)}] (**7**), [Cu(PPh_3_){Ph_2_B(3‐(CF_3_)Pz)_2_}] (**8**), [Cu(PPh_3_){PhB(3‐(CF_3_)Pz)_3_}] (**9**), [Cu(PPh_3_){Ph_3_B(6‐(CF_3_)Py)}] (**10**), [Cu(PPh_3_){Ph_2_B(6‐(CF_3_)Py)_2_}] (**11**) and [Cu(PPh_3_){PhB(6‐(CF_3_)Py)_3_}] (**12**) were fully characterized by multinuclear NMR spectroscopy, electrochemistry, and single‐crystal X‐ray diffraction. All compounds display trigonal‐planar copper centers, with mono(pyrazolyl)‐ and mono(pyridyl)borate complexes **7** and **10** exhibiting an unusual pseudo‐κ^2^ coordination supported by Cu•••C(ipso) interactions. Cyclic voltammetry revealed complex multielectron oxidation behavior, with poly(pyridyl)borate systems undergoing Cu(I)→Cu(II) oxidation at relatively lower potentials than their pyrazolyl analogues, consistent with stronger pyridyl σ‐donation. We also conducted efficacy tests on newly synthesized copper(I) complexes and related free ligands against a panel of human cancer cell lines from various solid tumors to explore relevant structure‐activity relationships. Studies on molecular target interaction, cellular redox modulation, and cell‐death pathways, have also been conducted to elucidate their mode(s) of action. Altogether, these results highlight how fluorinated scorpionate ligand design enable fine tuning of structure and properties of copper(I) complexes and underscore their promise as anticancer candidates.

## Graphical abstract

This study reports six new copper(I) complexes with unusual coordination and strong anticancer activity against a panel of human cancer cell lines derived from different solid tumors. The most active compounds outperform cisplatin, even in resistant cells, by inhibiting protein disulfide isomerase and inducing redox stress that triggers non‐apoptotic cell death consistent with paraptosis or cuproptosis.

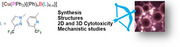

## 1 Introduction

Ligand design is essential for developing new metal complexes, as ligand steric, electronic, and architecture influence the properties of the resulting coordination compounds, thus playing a key role in advancing modern coordination chemistry. Poly(pyrazolyl)borates and related *N*‐scorpionate ligands [1] exemplify this well, and are among the most widely utilized ligands in coordination chemistry [2]. Over the past ∼60 years, their success has spurred the development of new scorpionates, which serve as excellent monoanionic supporting ligands for nearly all metals across the Periodic Table [2, 3, 4, 5, 6]. One key factor behind this success is the donor versatility achieved by modifying substituents on the boron center [7, 8, 9, 10, 11, 12, 13, 14, 15, 16, 17]. This includes the ability to easily tune the steric and electronic environment around the metal center by altering the heteroaromatic moieties or the substituents on the donor groups [3, 4, 18, 19, 20, 21, 22, 23].

The poly(2‐pyridyl)borate ligands are a relatively recent addition to the scorpionate family, are less developed, and differ significantly from the traditional poly(pyrazolyl)borate ligands [24]. Unlike poly(pyrazolyl)borates, where pyrazolyl groups are attached via B─N bonds that are somewhat susceptible to degradation through cleavage and rearrangement reactions [25, 26], poly(2‐pyridyl)borates feature pyridyl groups bound through less polar B─C bonds. Poly(2‐pyridyl)borates are better σ‐donating ligands than poly(pyrazolyl)borates [27]. They also present a different steric profile to the coordinated metal site, due to the involvement of six‐membered pyridyl donor arms instead of the five‐membered pyrazolyl moieties and have relatively more robust ligand backbone, attributable to less polar B─C linkages vs. B─N bonds. Given their high stability, strong donor ability toward metal ions, and tunability through various pyridyl derivatives, metal complexes with poly(2‐pyridyl)borates ligands are expected to exhibit notable differences in stability, solubility, optical spectra, and redox properties compared to their poly(pyrazolyl)borate counterparts [24, 27, 28]. In view of the popularity of poly(pyrazolyl)borates, the pyridyl versions are also bound to find growing utility in coordination chemistry [9, 27, 29, 30, 31, 32, 33, 34, 35, 36, 37, 38, 39, 40].

Our research activities in the field of scorpionates are focused on the development and applications of polyfluorinated or nitro‐substituted poly(pyrazolyl)borates [41, 42, 43, 44, 45] and other azolyl analogues like poly(triazolyl)borates [46, 47, 48, 49, 50]. The electron‐withdrawing (EWG) groups on these ligands reduce the electron density of metal centers, favoring low oxidation states and enhancing the metal electrophilicity [51]. Scorpionate ligands with EWG substituents are significantly weaker donors compared to the electron‐rich poly(pyrazolyl)borate analogues, displaying greater activity in certain catalytic processes like in the C─H and C–halogen bond functionalization chemistry via carbene insertion [52, 53].

The fluorinated versions of pyrazole based donors [54, 55, 56, 57], such as [HB(3,5‐(CF_3_)_2_Pz)_3_]^−^ (Pz = pyrazolyl), are particularly interesting and have enabled the isolation and detailed studies of rare molecules such as gold(I)‐carbonyl, silver(I)‐acetylene, gold(I)‐ethylene complexes [58, 59, 60], and useful in several biological applications, exhibiting promising anticancer and antimicrobial activities [61, 62].

It is also noteworthy that despite the extensive chemistry developed around bis‐ and tris‐(azolyl)borates, the mono(azolyl)borate analogues have received very little attention [63, 64, 65, 66, 67]. The trihydrido(pyrazolyl)borates [H_3_B(5‐(CF_3_)Pz)]Na and [H_3_B(3‐(NO_2_)Pz)]Na, represent two such examples [41, 45].

Considering the importance of fluorinated ligands in the above‐noted fields, our research activities have recently focused on the development and applications of the fluorinated versions of *B*‐aryl substituted poly(2‐pyridyl)borates [68, 69, 70, 71]. They include a recent report on the first fluorinated tris(2‐pyridyl)borate, [*t*‐BuC_6_H_4_B(6‐(CF_3_)Py)_3_]^−^ (Py = pyridyl), and its coinage metals ethylene chemistry [70]. In addition to the fluorinated *B*‐arylated poly(2‐pyridyl)borates, the *B*‐methylated analogues, [MeB(6‐(CF_3_)Py)_3_]^−^ and [Me_2_B(6‐(CF_3_)Py)_2_]^−^, have been prepared and utilized in coinage metal chemistry to compare the effect of changing the *B* substituent as well as to probe the interplay between κ^3^/κ^2^ complexes [72, 73].

**FIGURE 1:**
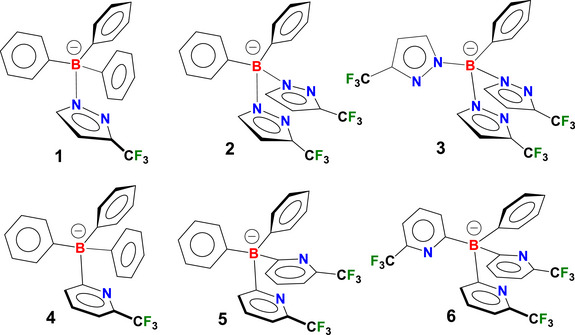
Structures of pyrazolyl‐ and pyridylborate‐ligands [Ph_3_B(3‐(CF_3_)Pz)]^−^ (**1**), [Ph_2_B(3‐(CF_3_)Pz)_2_]^−^ (**2**), [PhB(3‐(CF_3_)Pz)_3_]^−^ (**3**), [Ph_3_B(6‐(CF_3_)Py)]^−^ (**4**), [Ph_2_B(6‐(CF_3_)Py)_2_]^−^ (**5**), and [PhB(6‐(CF_3_)Py)_3_]^−^ (**6**) used in this work.

**FIGURE 2:**
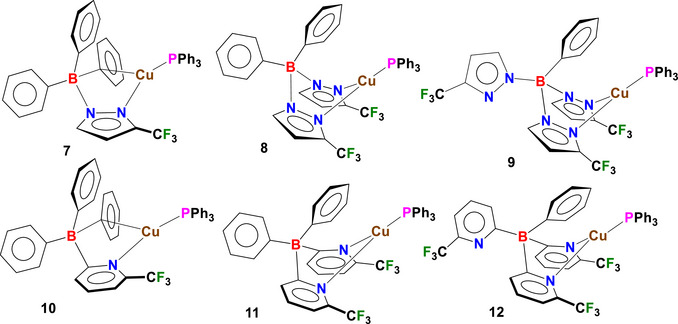
Structures of copper(I) phosphane complexes [Cu(PPh_3_){Ph_3_B(3‐(CF_3_)Pz)}] (**7**), [Cu(PPh_3_){Ph_2_B(3‐(CF_3_)Pz)_2_}] (**8**), [Cu(PPh_3_){PhB(3‐(CF_3_)Pz)_3_}] (**9**), [Cu(PPh_3_){Ph_3_B(6‐(CF_3_)Py)}] (**10**), [Cu(PPh_3_){Ph_2_B(6‐(CF_3_)Py)_2_}] (**11**) and [Cu(PPh_3_){PhB(6‐(CF_3_)Py)_3_}] (**12**) synthesized in this work.

As part of our ongoing investigations into the fluorinated poly(pyrazolyl)‐ and poly(2‐pyridyl)‐borates coinage metals coordination chemistry, here we describe the use of *B*‐phenylated *N*‐scorpionate type ligands [Ph_3_B(3‐(CF_3_)Pz)]^−^ (**1**), [Ph_2_B(3‐(CF_3_)Pz)_2_]^−^ (**2**), [PhB(3‐(CF_3_)Pz)_3_]^−^ (**3**), [Ph_3_B(6‐(CF_3_)Py)]^−^ (**4**), [Ph_2_B(6‐(CF_3_)Py)_2_]^−^ (**5**), and [PhB(6‐(CF_3_)Py)_3_]^−^ (**6**) containing electron withdrawing CF_3_ substituents on the pyrazolyl or pyridyl moieties (Figure 1) in the synthesis of six new copper(I) phosphane complexes [Cu(PPh_3_){Ph_3_B(3‐(CF_3_)Pz)}] (**7**), [Cu(PPh_3_){Ph_2_B(3‐(CF_3_)Pz)_2_}] (**8**), [Cu(PPh_3_){PhB(3‐(CF_3_)Pz)_3_}] (**9**), [Cu(PPh_3_){Ph_3_B(6‐(CF_3_)Py)}] (**10**), [Cu(PPh_3_){Ph_2_B(6‐(CF_3_)Py)_2_}] (**11**) and [Cu(PPh_3_){PhB(6‐(CF_3_)Py)_3_}] (**12**) (Figure 2). These ligands feature a variable number of phenyl groups attached to the boron atom and were selected to systematically modulate the steric and electronic properties of the resulting metal complexes. The complexes [Cu(PPh_3_){Ph_3_B(3‐(CF_3_)Pz)}] (**7**) and [Cu(PPh_3_){Ph_3_B(6‐(CF_3_)Py)}] (**10**) represent the first known examples of complexes featuring mono(pyrazolyl)‐ or mono(pyridyl)‐borate ligands with a pendant anionic triphenylborate group. Furthermore, fluorine‐containing compounds are of relevant interest in modern medicinal chemistry [74, 75, 76, 77, 78, 79, 80] as the introduction of fluorinated substituents in a molecule can induce substantial changes in its molecular, physicochemical, and biological properties [81, 82, 83].

In recent years, substantial progress has been achieved in exploring the anticancer potential of various scorpionate complexes, highlighting their significant cytotoxic activity across a range of cancer cell lines [84, 85]. Of particular interest are copper complexes, which have demonstrated promising properties as versatile, selective, and effective alternatives to conventional chemotherapeutic agents [86], thereby suggesting their potential to serve as the foundation for next‐generation anticancer therapies [87, 88, 89].

Motivated by the potential bioactivity of these new fluorinated copper(I) complexes, we also screened compounds **7–12** and their related free ligands against a panel of human cancer cell lines derived from various solid tumors, in order to explore relevant structure‐activity relationships.

## 2 Results and Discussion

### 2.1 Synthesis

**SCHEME 1:**
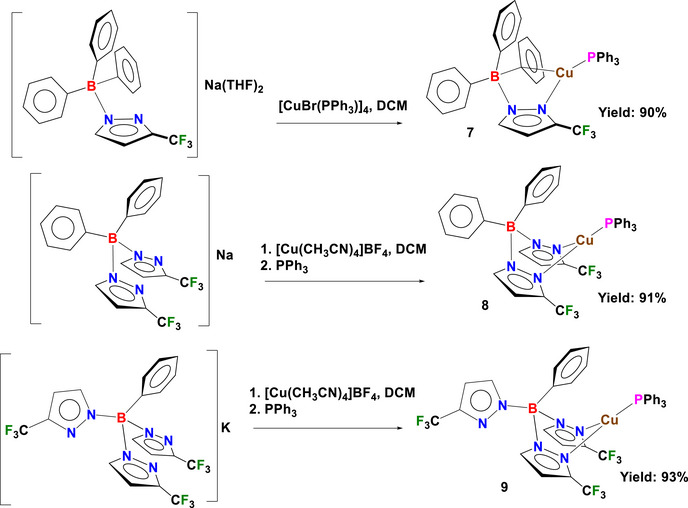
Synthesis of mono‐, bis‐, and tris‐(pyrazolyl)borate copper(I) phosphane complexes of **7–9**.

The synthesis of pyrazolylborate copper(I) phosphane complexes **7–9** is illustrated in Scheme 1. The complex [Cu(PPh_3_){Ph_3_B(3‐(CF_3_)Pz)}] (**7**) was synthesized by reacting [Ph_3_B(3‐(CF_3_)Pz)]Na(THF)_2_ with [CuBr(PPh_3_)]_4_ [90, 91] in anhydrous dichloromethane (DCM) and isolated in 90% yield. The analogous bis‐ and tris(pyrazolyl)borate complexes, [Cu(PPh_3_){Ph_2_B(3‐(CF_3_)Pz)_2_}] (**8**) and [Cu(PPh_3_){PhB(3‐(CF_3_)Pz)_3_}] (**9**), were prepared by treating their corresponding alkali metal salts, [Ph_2_B(3‐(CF_3_)Pz)_2_]Na and [PhB(3‐(CF_3_)Pz)_3_]K, with [Cu(CH_3_CN)_4_]BF_4_ in anhydrous DCM, followed by addition of PPh_3_, affording the desired products in 91% and 93%, respectively.

**SCHEME 2:**
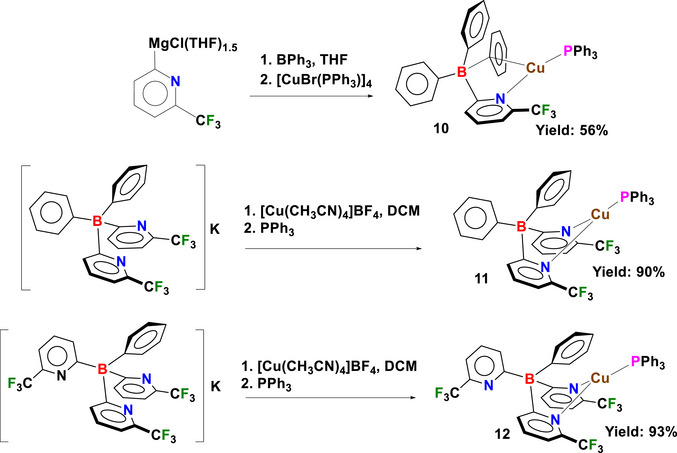
Synthesis of mono‐, bis‐, and tris‐(pyridyl)borate copper(I) phosphane complexes of **10–12**.

The synthesis of copper(I) phosphane complexes **10–12** supported by pyridylborates is shown in Scheme 2. The copper(I) complex [Cu(PPh_3_){Ph_3_B(6‐(CF_3_)Py)}] (**10**) was synthesized by adding [CuBr(PPh_3_)]_4_ to a premixed solution containing 6‐(CF_3_)‐2‐PyMgCl(THF)_1.5_ and BPh_3_ in anhydrous THF. It was isolated as in 56% yield. Although 6‐(CF_3_)‐2‐PyMgCl(THF)_1.5_ is a stable Grignard reagent when properly stored under an inert atmosphere and can be utilized for the synthesis of **10** as noted here, the use of the protonated ligand form of **4**, [Ph_3_B(6‐(CF_3_)Py)]H, may be more convenient. Several protonated poly(pyridyl)borate ligands have been successfully isolated and are generally quite stable under ambient atmosphere, easier to handle, and more readily available on a larger scale [29, 30, 33, 70, 71, 73]. An attempt to synthesize the protonated mono(pyridyl)borate ligand **4‐H** was made via aqueous workup of the reaction product between 6‐(CF_3_)‐2‐PyMgCl(THF)_1.5_ and BPh_3_. However, this attempt was unsuccessful and only resulted in the formation of 2‐(trifluoromethyl)pyridine, as confirmed by GC‐MS and NMR analyses. The bis‐ and tris‐(pyridyl)borate complexes, [Cu(PPh_3_){Ph_2_B(6‐(CF_3_)Py)_2_}] (**11**) and [Cu(PPh_3_){PhB(6‐(CF_3_)Py)_3_}] (**12**), were synthesized following a similar method as for **8** and **9**, yielding the desired products in 90% and 93%, respectively.

All six fluorinated copper(I) phosphane complexes (**7**–**12**) are white, air‐stable solids and can be stored at room temperature under ambient atmosphere for several months without noticeable decomposition.

### 2.2 Crystal Structures

**FIGURE 3:**
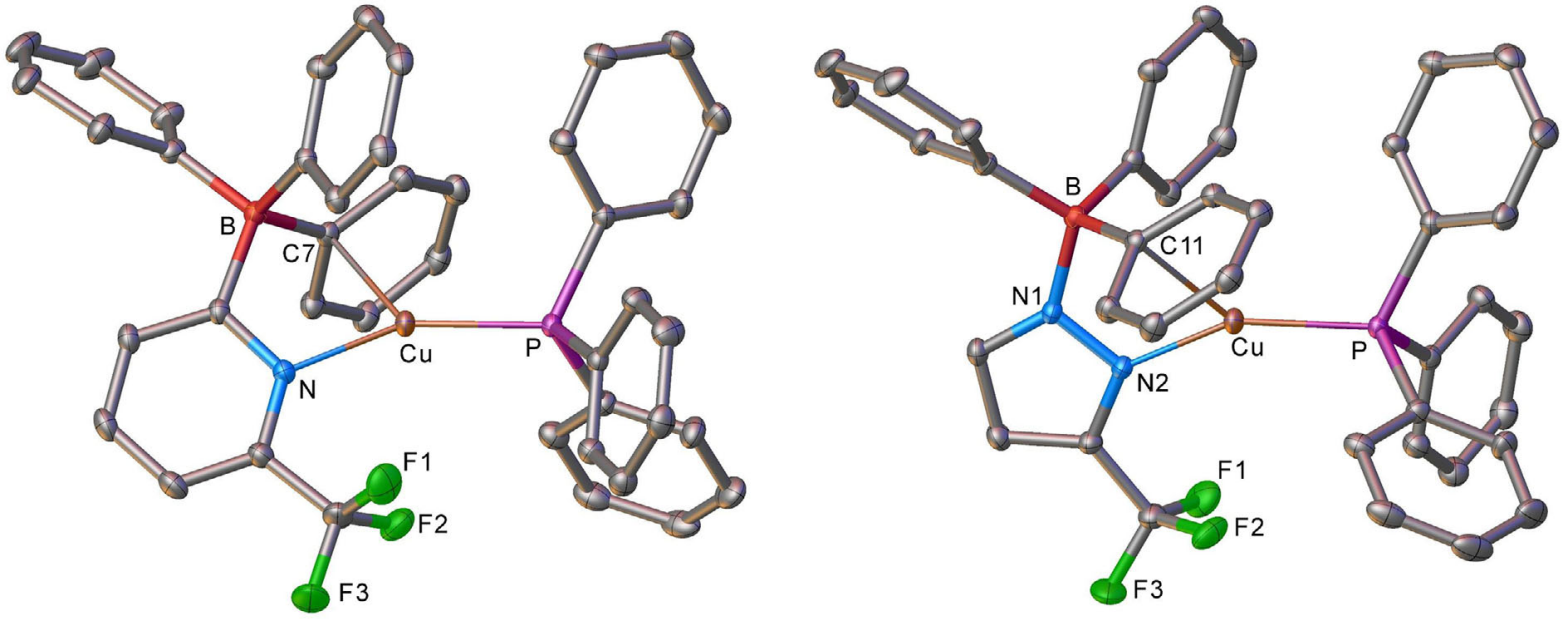
Crystal structures of [Cu(PPh_3_){Ph_3_B(3‐(CF_3_)Pz)}] (**7**, left) and [Cu(PPh_3_){Ph_3_B(6‐(CF_3_)Py)}] (**10**, right). Hydrogen atoms have been omitted for clarity.

The copper(I) phosphane complexes **7–12** yielded high‐quality single crystals suitable for X‐ray diffraction analysis. The structures were confirmed by X‐ray crystallography. Selected bond lengths and angles are summarized in Table 1. The X‐ray crystal structures of copper(I) phosphane complexes featuring mono(pyrazolyl)borate (**7**) and mono(pyridyl)borate (**10**) and are illustrated in Figure 3. Interestingly, instead of adopting the expected κ^1^‐mode of coordination via the single N‐donor, both complexes exhibit a pseudo κ^2^‐coordination mode (see Figure 3). They display an interaction between the copper centers and the *ipso* carbon of one of the phenyl rings on the borate ligand. The Cu•••C_(ipso)_ in compounds **7** and **10** are 2.3811(9) Å and 2.3559(10) Å, respectively (which is well within the Bondi's van der Waals contact separation of Cu and C of 3.10 Å). The Cu centers in **7** and **10** feature essentially trigonal‐planar coordination geometry, rather than the linear arrangement that uses only the donor nitrogen site of the scorpionate. The sum of all angles at Cu sites involving one nitrogen atom, C_(ipso)_ in the phenyl ring, and P atom are 359.7° in both cases, indicating that the Cu, N, C_(ipso)_, and P atoms sit in a same plane. Note that the linear coordination has been observed in monodentate metal complexes bearing sterically demanding ligands, such as [M(PPh_3_){4‐(Ph_3_B)‐2,6‐Trip_2_Py}] (M = Cu, Ag, Au) [92]. The N─Cu─P bond angles of **7** and **10** are 148.06(3)° and 151.40(3)°, respectively. The Cu─N bond lengths of the mono‐pyrazolyl and mono‐pyridyl borate complexes **7** and **10** are relatively shorter than the corresponding distances observed in **8**, **9**, and **11**, **12**. This is probably a result of lower steric effects of the mono(azolyl)borate ligand support in comparison to the related poly(azolyl)borate ligands.

**TABLE 1:** Selected bond distances [Å], angles [°] of compounds **7–12**. Cu•••C_(ipso)_ is the ipso‐carbon separation between the Cu and flanking phenyl group positioned above the Cu atom. Cu•••B is the nonbonded separation between the copper and boron atom. The ∑ at Cu represents the sum of all angles at Cu.

Parameter	7	8	9	10	11	12
Cu─P	2.1709 (3)	2.1642 (3)	2.1733 (4)	2.1899 (3)	2.2097 (12)	2.1978 (8)
Cu─N	1.9398 (8) 1.9398 (8)	2.0038 (10) 2.0102 (10)	2.0255 (10) 2.0132 (10)	1.9918 (8)	2.047 (3) 2.043 (3)	2.043 (2) 2.052 (2)
Cu•••C_(ipso)_	2.3811 (9)	2.9277 (11)	2.9841 (11)	2.3559 (10)	3.041 (4)	2.926 (2)
Cu•••B	2.8535 (9)	3.0567 (13)	3.0423 (12)	2.7898 (10)	3.045 (5)	2.985 (4)
∠N─Cu─P	148.06 (3)	133.27 (3) 134.59 (3)	131.29 (3) 137.11 (3)	151.40 (3)	139.83 (10) 128.06 (10)	137.88 (7) 129.01 (7)
∑ at Cu	359.7	360.0	360.0	359.7	360.0	360.0

**FIGURE 4:**
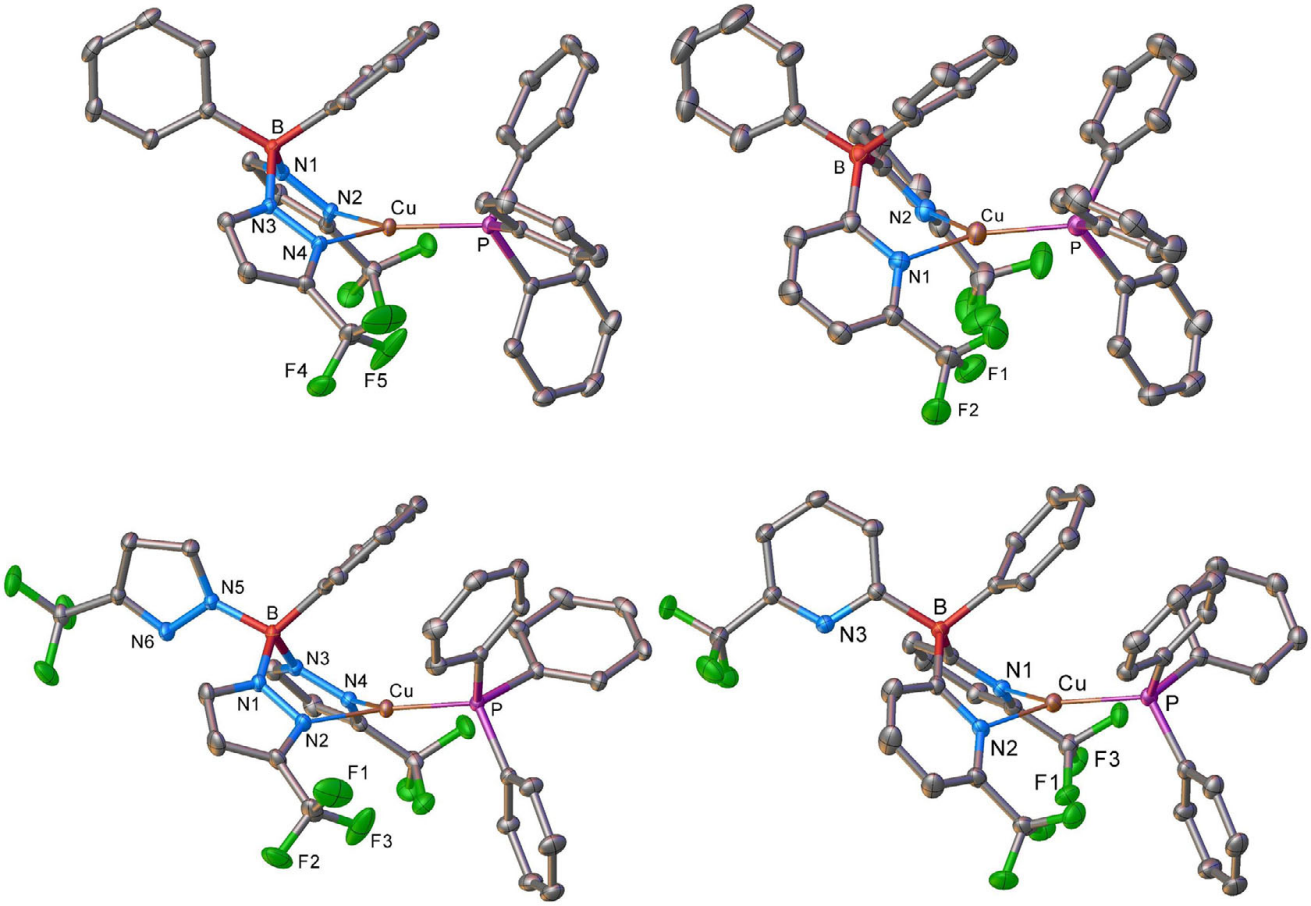
Crystal structures of [Cu(PPh_3_){Ph_2_B(3‐(CF_3_)Pz)_2_}] (**8**, top‐left), and [Cu(PPh_3_){PhB(3‐(CF_3_)Pz)_3_}] (**9**, bottom‐left), [Cu(PPh_3_){Ph_2_B(6‐(CF_3_)Py)_2_}] (**11**, top‐right), and [Cu(PPh_3_){PhB(6‐(CF_3_)Py)_3_}] (**12**, bottom‐right). Hydrogen atoms have been omitted for clarity.

As expected, the bis(pyrazolyl)borate ligand in [Cu(PPh_3_){Ph_2_B(3‐(CF_3_)Pz)_2_}] (**8**) and bis(pyridyl)borate ligand in [Cu(PPh_3_){Ph_2_B(6‐(CF_3_)Py)_2_}] (**11**) coordinate to the copper center through both nitrogen donor‐arms, forming bidentate chelates (Figure 4).

Although one of the flanking phenyl groups on boron is oriented above the metal site, there is no significant Cu•••C_(ipso)_ interaction. The Cu•••C_(ipso)_ distances in **8** and **11** are 3.057 Å and 3.045 Å, respectively. These values are close to the Bondi's van der Waals contact separation of 3.10 Å for Cu and C contact, but much longer than the typical Cu─C(sp^2^) covalent distance (2.05 Å) [93]. Notably, these weak interactions are not sufficient to induce pyramidalization at the copper centers, which remain planar, as evident from the sum of angles at copper (360.0°).

Tridentate, *B*‐phenylated scorpionate ligands have been reported to show remarkable coordination flexibility, adopting a variety of binding modes depending on the metal ion and ligand environment (see Figure S1). Interestingly, despite possessing three nitrogen donor arms, the tridentate ligands in complexes **9** and **12** give rise to essentially trigonal planar metal centers by coordinating through only two of the three nitrogen donor arms. In both cases, the scorpionate‐type ligands bind to copper sites in bidentate fashion in solid state, with the *B*‐phenyl group oriented above the metal center in place of the third N‐containing donor arm (see Figure 4). This coordination behavior has been previously observed in related phenylated borate chelator complexes such as [Ag(C_2_H_4_){PhB(3‐(CF_3_)Pz)_3_}], [PhB(3‐(Fc)Pz)_3_]Tl, [Cu(CNBu*^t^*){*t*‐BuC_6_H_4_B(6‐(CF_3_)Py)_3_}] [71, 94, 95]. In contrast, other phenylated borate complexes have been reported to utilize all three nitrogen donor arms to chelate the metal center, as seen in [Cu(CO){PhB(3‐(CF_3_)Pz)_3_}], [PhB(3‐(*^t^* Bu)Pz)_3_]Li, [PhB(6‐(Me)Py)_3_]NiBr [32, 71, 96]. Additional coordination mode can also be seen where only two donor arms are coordinated, and the third nitrogen donor arm is positioned parallel and above the metal center, as seen in [PhB(3‐(*^t^* Bu)Pz)_3_]Tl [97]. These examples collectively highlight the versatility of tridentate phenylated borate ligands in coordination geometries through selective engagement of donor arms. The Cu─N and Cu─P bond distances of **8** and **9** are somewhat shorter compared to the analogous distance in the related poly(pyridyl)borate adducts **11** and **12**. This is probably a result of lower steric demands of the poly(pyrazolyl)borate ligand support in comparison to the related poly(pyridyl)borate ligand [27].

### 2.3 NMR Spectroscopy

The formation of the six complexes was confirmed by comparing the chemical shifts in their ^1^H NMR spectra with those of the corresponding free ligands. More interestingly, through‐space coupling between phosphorus and fluorine nuclei (*J*_P‐F_) was observed in some of the fluorinated copper(I) phosphane complexes. The magnitude of this coupling constant provides insight into the spatial proximity of the P and F atoms, offering complementary information to that obtained from X‐ray crystal structures. Due to the dynamic rotation of the CF_3_ group in solution, accurately estimating the closest nonbonded intramolecular P···F distance from solid‐state structures is challenging. However, the distance between the phosphorus atom and the carbon atom of the CF_3_ group is more readily measured and still provides useful information about P···F through‐space interactions in solution. The ^19^F─^31^P coupling constants observed in compounds **7–12**, along with values from previously reported fluorinated copper(I) triphenylphosphane complexes, are summarized in Table S1. Overall, the poly(pyridyl)borate complexes (**10**–**12**) exhibit larger coupling constants than poly(pyrazolyl)borate analogues described in this work and prior studies, with the highest value of 11.4 Hz observed for [Cu(PPh_3_){PhB(6‐(CF_3_)Py)_3_}] (**12**). This larger coupling can be attributed to the closer proximity of the CF_3_ substituent on the pyridyl ring (at the 6‐position) compared to that on the pyrazolyl ring (3‐position), bringing the fluorine nuclei closer to the metal center and to the phosphorous center. Interestingly, the ^19^F NMR spectrum of compound **12** in CDCl_3_ displayed two doublet signals at room temperature with ^19^F─^31^P coupling constant of 11.1 Hz, and one with a smaller coupling constant value of 5.8 Hz and a singlet (Figure S31). As discussed above, the tridentate phenylated borate ligands could display different coordination modes (see also Figure S1). The larger ^19^F─^31^P coupling may arise from the isomer observed in the solid state, similar to that of **11** with the phenyl group flanking the copper site, while the smaller coupling may be from an isomer having a flanking pyridyl moiety and fluxionality between the three pyridyl donor sites in solution at room temperature. At −20°C, compound **12** exists as only one isomer, similar to that observed in the solid state, resulting in a doublet (for CF_3_ moieties of pyridyl groups attached to the copper site) and a singlet (for the CF_3_ groups of the pyridyl group occupying the axial position away from the copper center) in the ^19^F NMR spectrum in CD_2_Cl_2_ (Figure S33). The ^31^P NMR spectrum of the copper complex **7** shows a sharp singlet, consistent with the absence of through‐space interaction between P and F atoms, while complexes **8–12** displayed broader signals, likely due to unresolved coupling between the phosphorous and the nearby CF_3_‐fluorines. The ^31^P NMR chemical shifts of the mono(pyrazolyl)‐ and mono(pyridyl)‐borate complexes **7** and **10** appear further downfield compared to their bis‐and tris‐substituted analogues.

### 2.4 Electrochemistry

Comprehensive electrochemical studies were conducted to probe metal‐ligand interactions and electronic effects in the copper complexes investigated in this work. Specifically, cyclic voltammetry experiments were performed to elucidate the redox cycling of copper in the Cu(I) → Cu(II) → Cu(I) oxidation states. All the copper complexes were prepared in acetonitrile, using TBAPF_6_ as the supporting electrolyte. The cell configuration comprised a glassy carbon working electrode, a platinum wire counter electrode, and a non‐aqueous Ag^+^/Ag reference electrode (E = +0.80 V vs. NHE). Ferrocene was incorporated as an internal standard to allow for precise calibration of the redox potentials. To prevent interference from dissolved oxygen and moisture, the electrolyte solutions were thoroughly purged with nitrogen gas before measurement. This setup enabled an accurate assessment of the Cu(I)/Cu(II) interconversion and provided deeper insights into the underlying metal‐ligand interactions.

Figure S38 shows the cyclic voltammograms of the poly(pyrazolyl)borate ligand‐supported copper(I) complexes. Compound [Cu(PPh_3_){Ph_3_B(3‐(CF_3_)Pz)}] (**7**) exhibits two oxidation peaks at 0.90 and 1.39 V, accompanied by a weak and poorly resolved reduction feature on the reverse scan. The latter oxidative shoulder at higher positive potential may arise from ligand‐centered oxidation, although a Cu(II)/Cu(III) process cannot be ruled out, consistent with prior reports [98, 99]. Notably, this oxidative response occurs well beyond, and at much higher potential than, the oxidation of the ligand in the related sodium salt at 0.98 V (Figure S39a). [Cu(PPh_3_){Ph_2_B(3‐(CF_3_)Pz)_2_}] (**8**) displays three anodic processes at 0.88, 1.06, and 1.31 V, together with a single reduction peak at −0.059 V. The tris(pyrazolyl)borate [Cu(PPh_3_){PhB(3‐(CF_3_)Pz)_3_}] (**9**) complex shows three oxidative transitions at 0.78, 0.85, and 0.98 V, as well as a reduction at 0.15 V. A slower scan rate was employed for **9** to obtain well‐defined redox features, as the steric bulk of the ligand framework leads to hindered diffusion and peak broadening under faster scan conditions [100].

For comparison, the highly fluorinated analogue [Cu(PPh_3_){HB(3,5‐(CF_3_)_2_Pz)_3_}] and [Cu(NCCH_3_){HB(3,5‐(CF_3_)_2_Pz)_3_}] reported in the literature display a single irreversible oxidation near 1.50 V, with a reduction peak at 0.5 V under similar conditions in the cathodic sweep [101]. The nonfluorinated [Cu(PPh_3_){HB(3,5‐(CH_3_)_2_Pz)_3_}] displays an anodic wave corresponding to Cu(I) → Cu(II) process around 1.0 V. In addition, a series of Cu(I) complexes containing 3‐*tert‐*butyl and 3,5‐diphenyl substituents on the tris(pyrazolyl)borate ligand had oxidation potentials varying from ca. 0.70 V to ca. 1.0 V [102]. To understand the observed electrochemistry further, we have also conducted cyclic voltammetry measurements on alkali metal salts of the same ligands (Figure S39). The [Ph_3_B(3‐(CF_3_)Pz)]Na(THF)_2_ exhibits an oxidation peak at 0.98 V, while no reduction event was observed during the cathodic potential sweep. A similar feature was also observed for [Ph_2_B(3‐(CF_3_)Pz)_2_]Na, which exhibited a single oxidation process at 1.22 V, with no reduction detected during the cathodic scan. In contrast, no redox activity was observed for [PhB(3‐(CF_3_)Pz)_3_]K across the applied potential window. Based on these comparisons, the first anodic waves at 0.90 V, 0.88 V, and 0.78 V of **7, 8**, and **9** may be tentatively assigned to the Cu(I) → Cu(II) oxidation. Unfortunately, the additional redox events, overlapping ligand and metal oxidation potentials, and likely conformational changes during the oxidation, prevent a more definitive assignment. Conformational reorganizations and potential complications in such studies have been reported previously [102, 103, 104].

Figure S40 shows cyclic voltammograms of the poly(pyridyl)borate containing Cu(I) complexes, which also revealed multielectron oxidative behavior. The mono(pyridyl)borate derivative [Cu(PPh_3_){Ph_3_B(6‐(CF_3_)Py)}] (**10**) displays oxidation waves at 0.64 and 1.20 V, together with a reduction event at 0.26 V. A similar feature of Cu(II) → Cu(III) oxidation was observed as in the case of **7**, and the more defined peak in **10** could be resulted from the electron rich Cu^2+^ center. The bis(pyridyl)borate complex [Cu(PPh_3_){Ph_2_B(6‐(CF_3_)Py)_2_}] (**11**) shows two anodic processes at 0.52 V and 0.88 V, accompanied by a single reduction peak at −0.10 V. The tris(pyridyl)borate analogue [Cu(PPh_3_){PhB(6‐(CF_3_)Py)_3_}] (**12**) presents oxidation waves at 0.52 and 0.85 V, and a reduction peak at −0.076 V. The copper‐free ligands were examined independently to establish their intrinsic electrochemical profiles. [Ph_2_B(6‐(CF_3_)_2_Py)_2_]H exhibits two oxidation events at 0.97 and 1.13 V (Figure S41a). In contrast, the protonated tris(pyridyl)borate ligand ([PhB(6‐(CF_3_)_2_Py)_3_]H) showed only one defined oxidation peak at 0.98 V under similar conditions employed (Figure S41b). Based on these observations, the first anodic waves observed at 0.64, 0.52 and 0.52 V for **10**, **11**, and **12**, respectively, can be assigned to the Cu(I) → Cu(II) oxidation while the subsequent higher‐potential features are likely associated with ligand‐based oxidations, although metal centered oxidations resulting from different conformations cannot be completely ruled out. In the observed voltammograms for these pyridyl‐ligand systems, a significant decrease in the current density was observed, which possibly could result from increased steric bulk and reduced diffusivity of the ligand [100]. Overall, a clear ligand‐dependent effect on the oxidation behavior of the copper(I) center is observed when comparing the poly(pyrazolyl)borate and poly(pyridyl)borate series. The poly(pyridyl)borate ligands, being the more basic donors [30], stabilize higher electron density at the metal center and consequently facilitate oxidation at lower potentials. As a result, the poly(pyridyl)borate‐supported Cu(I) complexes consistently exhibit anodic processes at lower potentials relative to their poly(pyrazolyl)borate counterparts. The compounds **8** and **9** undergo Cu(I)/Cu(II) oxidation at lower potentials than the mono(pyrazolyl)borate complex **7**, a trend mirrored in the corresponding pyridyl complexes **11** and **12** vs **10**. This difference arises from the ligand coordination environment; the tris‐ and bis‐ligated systems provide two *N*‐based donor arms to the copper center, increasing electron density and stabilizing the oxidized species. In contrast, mono‐ligated complexes offer only one *N*‐donor arm, resulting in a less electron‐rich metal center that requires higher potentials for Cu(I)/Cu(II) oxidation [104, 105, 106, 107].

### 2.5 Biological Studies

The novel copper(I) complexes **7–12** and the copper‐free ligands **1–3, 5, 6** (ligand **4** was not isolated and therefore not included in the biological evaluation) were tested for their cytotoxic activity toward a series of human cancer cell lines in bidimensional (2D) assays. In particular, the in‐house cancer cell line panel contains examples of human colon (HCT‐15), lung (A549), breast (MCF‐7), pancreatic (PSN‐1), germinal (NTERA‐2) and ovarian (2008) cancers. As reported in the Experimental Section, cisplatin was selected as the reference metallodrug and was evaluated under the same experimental conditions. The cytotoxicity parameters obtained by means of the MTT assay after 72 h of drug exposure, and expressed in terms of IC_50_, are listed in Table 2.

**TABLE 2:** Cytotoxic activity evaluated by the MTT test at 72 h. IC_50_ values were calculated with a four‐parameter logistic model (*p* < 0.05). R.F. = IC_50_ (resistant subline)/IC_50_ (wild‐type cells). S.D: Standard deviation.

	IC_50_ (µM) ± S.D.						
	HCT‐15	A549	MCF‐7	PSN‐1	NTERA‐2	2008	C13*
**7**	6.7 ± 0.3	9.3 ± 1.1	8.6 ± 1.6	7.5 ± 1.0	6.9 ± 0.3	7.9 ± 1.1	8.6 ± 1.2 (1.1)
**8**	20.1 ± 1.7	22.4 ± 2.5	23.3 ± 0.8	21.3 ± 2.2	23.5 ± 2.3	20.9 ± 3.5	25.4 ± 2.8 (1.2)
**9**	11.5 ± 0.8	15.7 ± 3.4	14.1 ± 0.4	13.3 ± 1.1	12.1 ± 1.3	12.6 ± 0.8	15.7 ± 1.2 (1.3)
**10**	8.4 ± 0.4	9.6 ± 0.7	8.1 ± 1.2	9.8 ± 1.3	10.3 ± 3.1	8.9 ± 1.2	9.3 ± 1.5 (1.1)
**11**	17.1 ± 2.2	20.2 ± 2.1	17.8 ± 2.2	18.4 ± 2.6	20.8 ± 2.9	22.5 ± 2.9	27.8 ± 3.2 (1.2)
**12**	4.6 ± 0.6	6.6 ± 0.8	5.0 ± 0.5	5.8 ± 1.0	4.9 ± 0.8	5.2 ± 0.4	5.5 ± 0.8 (1.1)
**1‐Na(THF)_2_**	>50	>50	>50	>50	>50	>50	−
**2‐Na**	>50	>50	>50	>50	>50	>50	−
**3‐K**	>50	>50	>50	>50	>50	>50	−
**5‐H**	>50	>50	>50	>50	>50	>50	−
**6‐H**	>50	>50	>50	>50	>50	>50	
**cisplatin**	13.9 ± 1.6	8.4 ± 0.9	6.9 ± 1.1	12.1 ± 2.8	10.2 ± 2.0	2.1 ± 1.1	28.7 ± 2.4 (13.7)

Overall, the 2D cytotoxicity studies highlighted that all copper(I) complexes elicited IC_50_ values in the micromolar range and showed a comparable pattern of response across the six tumor cell lines. Within the pyrazolyl‐based series, the mono‐pyrazolyl borate complex **[Cu(PPh_3_){Ph_3_B(3‐(CF_3_)Pz)}] (7)** proved to be the most effective derivative, with an average IC_50_ value of 7.8 µM, whereas the bis‐pyrazolyl derivative **8** elicited the weakest cytotoxic activity, with an average IC_50_ value of 21.9 µM. On the other hand, for the analogous pyridyl complexes **10–12**, the in vitro antitumor profile followed the order **12**> **10**> **11**. Notably, the tris‐pyridyl complex **[Cu(PPh_3_){PhB(6‐(CF_3_)Py)_3_}] (12)** exhibited a cytotoxic potency on average 2.5 times greater than that of the reference metallodrug cisplatin, representing the most effective complex among all the newly developed ones.

The antiproliferative activity of the novel copper(I) compounds was also investigated in a cisplatin‐resistant cancer cell subline (C13* cells). All tested complexes exhibited a very similar activity level in both the cisplatin‐sensitive (2008) and ‐resistant (C13*) cell lines. The resulting resistance factor (RF) values indicate that these compounds are capable of overcoming cisplatin resistance.

The noteworthy cytotoxic efficacy shown by the newly developed copper(I) complexes in 2D monolayer cultures was further validated using suitably established 3D cell culture models. Actually, 3D spheroid cell culture systems are widely recognized to better reproduce the cancer in vivo microenvironment, both in terms of physio‐pathological characteristics (*e.g*., gene expression, cell‐cell interactions and metabolism) and drug‐related interactions (drug permeation, retention and intracellular trafficking), making them more predictive models for anticancer drug screening [108]. Table 3 summarizes the IC_50_ values obtained after treatment of 3D cell spheroids derived from human colon cancer cells with complexes **7–12**, alongside cisplatin as the reference drug.

**TABLE 3:** Spheroids from HCT‐15 cells were treated for 72 h with increasing concentrations of tested compounds. The growth inhibitory effect was evaluated by means of the APH assay. IC_50_ values were calculated from the dose–Survival curves by the four‐parameter logistic model (*p* < 0.05). S.D. = standard deviation.

Compound	IC_50_ (µM) ± S.D.
	HCT‐15
**7**	34.5 ± 2.6
**8**	75.2 ± 8.6
**9**	56.7 ± 4.1
**10**	88.5 ± 1.4
11	102.3 ± 12.1
12	35.6 ± 2.3
Cisplatin	68.2 ± 4.6

Coherently with 2D cell‐based findings, compounds **7** and **12** displayed the highest antitumor potential in 3D models, with IC_50_ values about 2‐fold lower than that of cisplatin. Interestingly, also the tris‐pyrazolyl derivative **9** showed a cytotoxic potency higher compared to that of cisplatin.

### 2.6 Mechanistic Studies

Over the past three decades, numerous mechanistic investigations on copper(II) complexes have identified a variety of molecular targets; among them, protein disulfide isomerase (PDI) has recently been emerged as a key target for several copper(I) and copper(II) complexes [109, 110]. On this basis, we also evaluated the ability of the newly developed copper(I) complexes to act as PDI inhibitors. The PDI enzyme was treated with increasing concentrations (range 1–100 µM) of tested complexes, and the ability to hamper its activity was assessed using a biochemical colorimetric assay (Proteostat kit). As shown in Table 4, copper derivatives **7–12** elicited IC_50_ values in the micromolar range and were significantly lower than that of bacitracin, a well‐known PDI inhibitor, used as a reference compound. It is worth noting that complexes **7** and **12** were the most effective derivatives, showing inhibition IC_50_ values that were 24‐ and 27‐fold lower, respectively, than that of bacitracin.

**TABLE 4:** PDI inhibition induced by **7–12** was measured by Proteostat PDI assay kit. The PDI inhibitor bacitracine (range 100–1000 µM) was used as a positive control.

Compound	IC_50_ (µM) ± S.D.
**7**	16.8 ± 3.1
**8**	46.3 ± 4.4
**9**	27.3 ± 3.1
**10**	84.3 ± 5.4
**11**	27.2 ± 2.1
**12**	21.3 ± 0.9
Bacitracin	570 ± 12

**FIGURE 5:**
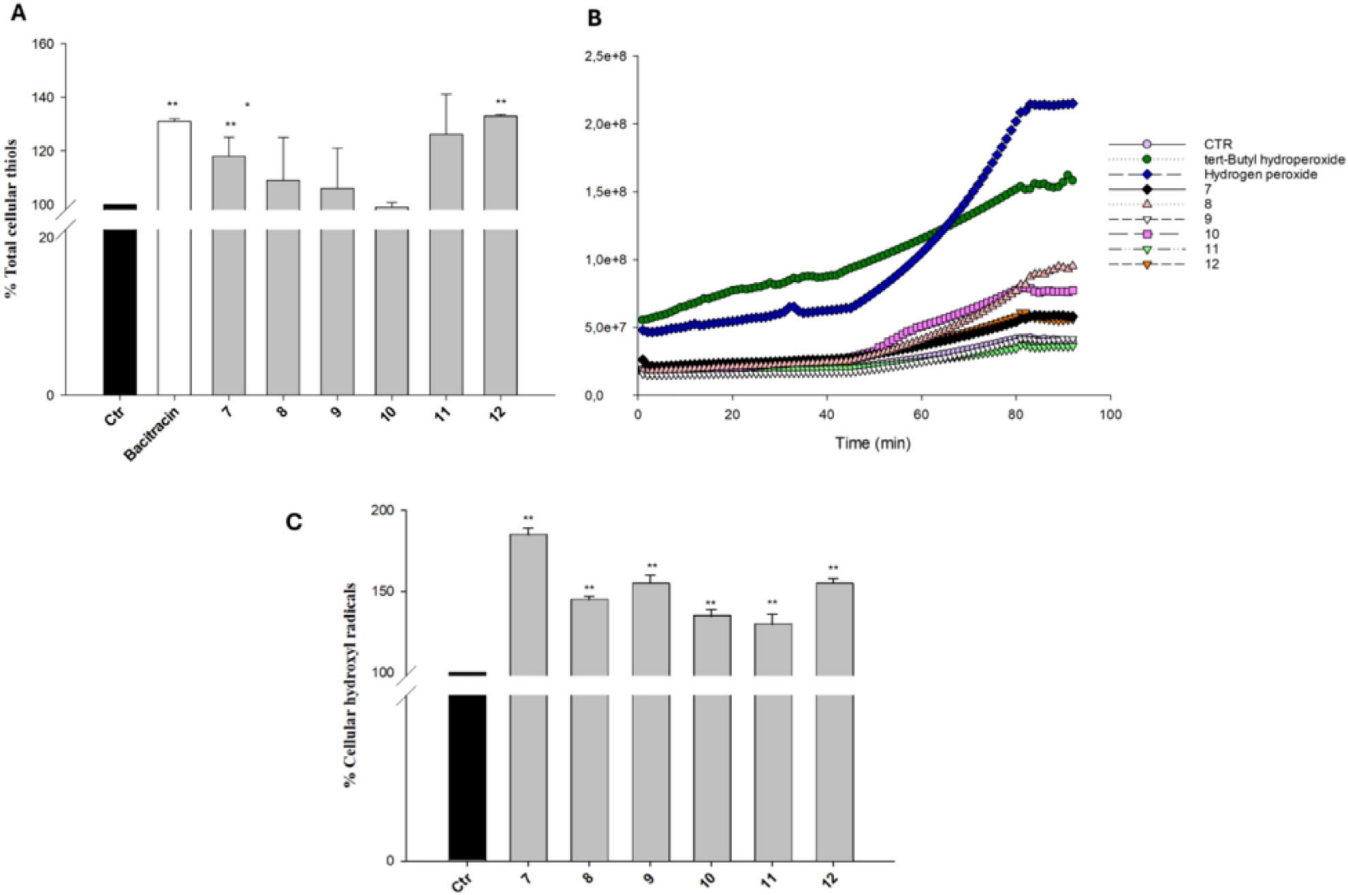
(A) Sulfhydryl content in HCT‐15 cancer cells incubated for 36 h with 5 µM of tested compounds or bacitracin (0.5 mM). The sulfhydryl group amount was determined by the DTNB assay. Error bars indicate S.D. **p < 0.01 compared with control. Hydrogen peroxide (B) or hydroxyl radical (C) cellular levels. HCT‐15 cells were preincubated in PBS/10 mM glucose medium for 20 min at 37°C in the presence of 10 µM CM‐H_2_DCFDA (B) or fluorescent probe RH‐EDA (C) and then treated with the tested compounds. In C, error bars indicate S.D. **p < 0.01 compared with control.

Given that a primary cellular role of PDI is to catalyze disulfide‐bond reduction and thiol oxidation, we next quantified the levels of reduced thiols in HCT‐15 colon cancer cells exposed to complexes **7–12**. (Figure 5A). Remarkably, cells treated for 36 h with 5 µM of the tested complexes showed a great increase in total sulfhydryl content, which reached up to 130% in cells treated with complex **12**.

It is well known that copper(I) reacts with hydrogen peroxide in a Fenton‐like process that both consumes hydrogen peroxide and forms reactive hydroxyl radicals (OH). These radicals are profoundly cytotoxic to cancer cells and can induce severe oxidative stress, resulting in extensive damage DNA, proteins, and lipids, ultimately promoting cell‐death pathways.

Accordingly, HCT‐15 cancer cells following treatment with tested copper(I) complexes showed very low levels of hydrogen peroxide (Figure 5B), comparable to or lower than those measured in control cells, alongside significantly high cellular levels of **^∙^** OH (Figure 5C).

**FIGURE 6:**
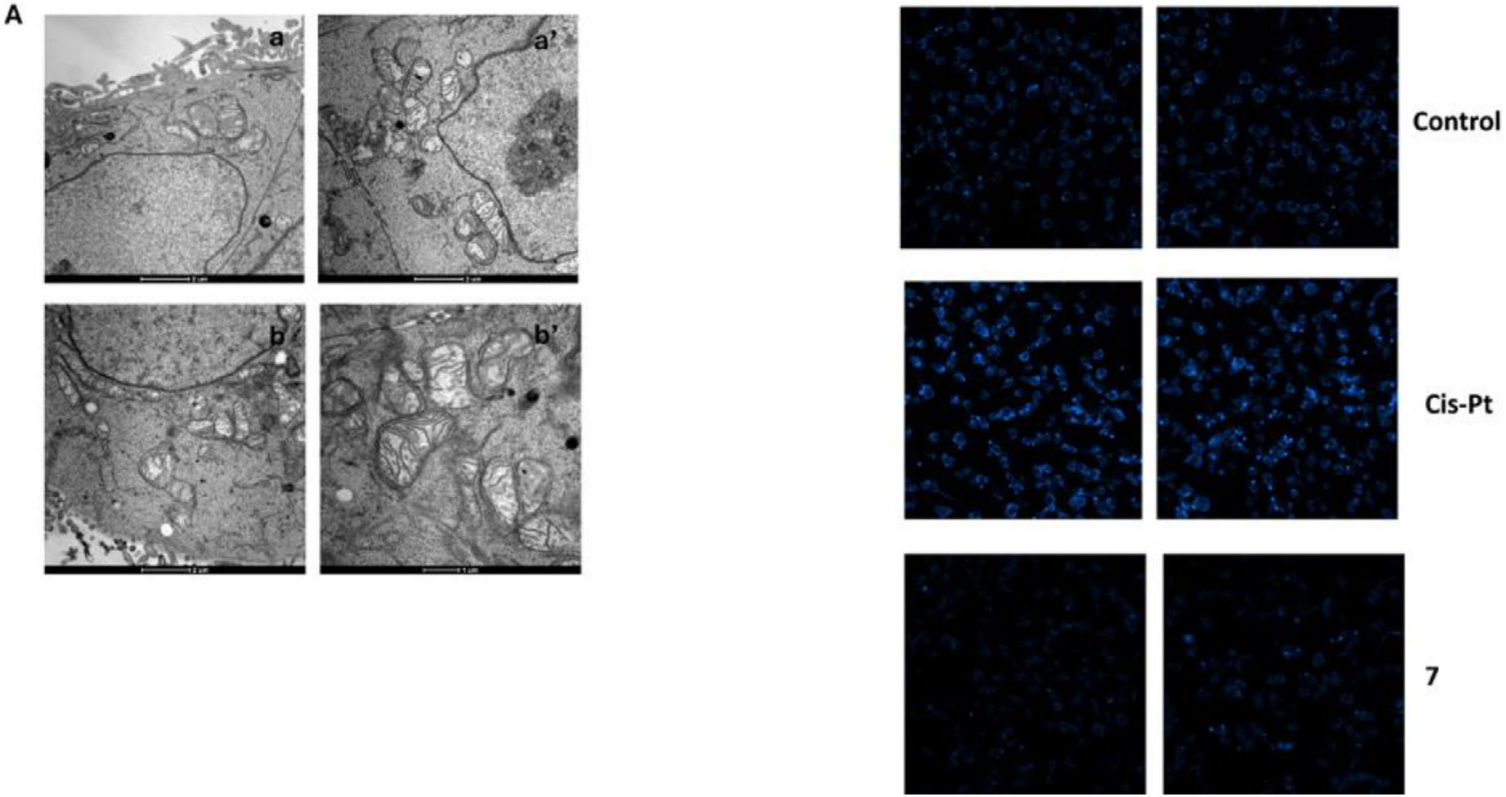
Morphological analysis. (A) TEM analysis of HCT‐15 colon cancer cells untreated (a and a’) or treated (b and b’) for 24 h with IC_50_ of complex **7**. (B) Hoechst staining of untreated (Control) HCT‐15 cells or incubated for 48 h with IC_50_ doses of cisplatin or complex **7** (10 × magnification).

Detailed morphological analysis by transmission electron microscopy (TEM) (Figure 6B) showed that exposing HCT‐15 cells to IC_50_ concentrations of complex **7** for 24 h caused a pronounced enlargement of the ER cisternae, a clear ultrastructural hallmark of ER stress, along with marked cytoplasmic vacuolization. In addition, a modest increase in mitochondrial size (swelling) was observed, accompanied by reduced electron density in the inner membrane and matrix regions, as well as alterations in cristae architecture. In contrast, no signs of nuclear damage, chromatin condensation, or cell shrinkage were detected. Consistent with these observations, HCT‐15 cells treated for 48 h with the IC_50_ of complex **7** and stained with the Hoechst 33342 probe displayed none of the classical morphological hallmarks of apoptosis (such as brightly stained nuclei, chromatin condensation, fragmentation), which were clearly evident in colorectal cancer cells exposed to the reference metallodrug cisplatin (Figure 6B). Taken together, these findings support the activation of a non‐apoptotic cancer cell‐death pathway, consistent with paraptosis or, as more recently defined, cuproptosis [111].

## 3 Conclusions

This work demonstrates that *B*‐phenylated fluorinated poly(pyrazolyl)‐ and poly(2‐pyridyl)borate ligands provide a versatile platform for tuning the coordination geometry, redox behavior, and biological activity of copper(I) phosphane complexes. Solid‐state structures revealed that the mono(pyrazolyl)‐ and mono(pyridyl)‐borate complexes **[Cu(PPh_3_){Ph_3_B(3‐(CF_3_)Pz)}] (7)** and **[Cu(PPh_3_){Ph_3_B(6‐(CF_3_)Py)}] (10)** adopt an unusual pseudo‐κ^2^ coordination mode involving the azolyl moiety and a secondary Cu···C(ipso) interaction, influencing both the steric environment and the electron density at the metal center. The bis‐, and tris‐(pyrazolyl/pyridyl)borate ligand supported copper complexes **8–9** and **11–12** feature trigonal planar metal sites and *κ^2^‐NN*‐bound scorpionates. Biological studies demonstrated that all copper(I) complexes exert micromolar cytotoxicity across several human cancer cell lines, with **[Cu(PPh_3_){Ph_3_B(3‐(CF_3_)Pz)}] (7)** and **[Cu(PPh_3_){PhB(6‐(CF_3_)Py)_3_}] (12)** emerging as the most potent derivatives in both 2D and 3D cell culture models. Notably, their cytotoxic activity surpasses that of cisplatin and remains effective in cisplatin‐resistant cancer cells, underscoring the therapeutic relevance of copper‐based agents in overcoming platinum resistance. Unfortunately, the available data do not yet allow the definition of clear structure‐activity relationships. In fact, compounds **7** and **12**, belong to distinct ligand families within the series (pyrazolyl‐ and pyridyl‐based scaffolds), suggesting that their enhanced cytotoxic profile results from a combination of structural and electronic factors, rather than from a single dominant substituent effect. This complexity is consistent with the multifactorial contributions of ligand denticity, steric encumbrance, Cu···C secondary interactions, and the presence of fluorinated azolyl groups, all of which may jointly influence copper(I) reactivity and biological performance. Mechanistic investigations indicated that these complexes act as efficient inhibitors of PDI and induce a profound perturbation of intracellular redox homeostasis, as shown by elevated sulfhydryl content, depletion of hydrogen peroxide, and increased hydroxyl radical formation. Ultrastructural analyses further suggest that the resulting cell death does not follow classical apoptotic routes but instead aligns with non‐apoptotic processes consistent with paraptosis or emerging copper‐dependent pathways such as cuproptosis. Their ability to inhibit PDI, disrupt redox balance, and trigger non‐apoptotic cell death pathways underscores the originality of this class of compounds and its potential to address chemoresistance. Overall, this study underscores the value of fluorinated scorpionate ligand frameworks in enabling fine control over the structural and electron‐transfer properties of copper(I) complexes, while simultaneously producing bioactive compounds with promising anticancer profiles. The strong interplay between ligand structure, redox activity, and biological function lays the groundwork for creating innovative anticancer metallodrugs.

## 4 Experimental Section

### 4.1 Chemistry

#### 4.1.1 Materials and General Methods

All preparations and manipulations were carried out under an atmosphere of purified nitrogen using standard Schlenk techniques or in an MBraun drybox equipped with a −25°C refrigerator. Dichloromethane and hexane were dried by passing HPLC‐grade solvent through a Solvent Purification System (SPS, Innovative Technologies Inc.) and stored in Straus flasks. Tetrahydrofuran was distilled from a sodium/ketyl still. Glassware was oven‐dried overnight at 150°C. Solvents were purchased from commercial sources and purified before use. NMR spectra were recorded at 25°C on a JEOL Eclipse 400 spectrometer (^1^H, 400 MHz; ^13^C, 101 MHz, ^19^F, 376 MHz; ^11^B, 128 MHz and ^31^P, 162 MHz), or a JEOL Eclipse 500 (^1^H, 500 MHz; ^13^C, 126 MHz; ^19^F, 471 MHz; ^11^B, 160 MHz and ^31^P, 202 MHz), and all the spectral data were processed on MNova. ^1^H and ^13^C{^1^H} NMR spectra are referenced to the solvent peak (^1^H, CDCl_3_ δ 7.26, CD_2_Cl_2_ δ 5.32; ^13^C, CDCl_3_ δ 77.16, CD_2_Cl_2_ δ 54.00). ^19^F NMR values were referenced to external CFCl_3_. ^11^B{^1^H} NMR values were referenced to external 15% BF_3_.OEt_2_ in CDCl_3_. ^31^P{^1^H} NMR values were referenced to external 85% H_3_PO_4_. ^1^H, ^13^C{^1^H}, ^19^F, ^11^B{^1^H}, and ^31^P{^1^H} NMR chemical shifts are reported in ppm and coupling constants (J) are reported in Hertz (Hz). Abbreviations used for signal assignments: Py = pyridyl, Ph = phenyl, Pz = pyrazole, s = singlet, d = doublet, dd = doublet of doublets, t = triplet, q = quartet, pent = pentet, m = multiplet, br = broad, br s = broad singlet, * = peak belongs to the less abundant isomer. Elemental analyses were performed using a Perkin–Elmer Model 2400 CHN analyzer. High‐resolution (HR) mass spectra were recorded at the Shimadzu Center Laboratory for Biological Mass Spectrometry at UTA. [Ph_2_B(3‐(CF_3_)Pz)_2_]Na [54], [PhB(3‐(CF_3_)Pz)_3_]K [94], 6‐(CF_3_)‐2‐PyMgCl(THF)_1.5_ [70, 73], [Ph_2_B(6‐(CF_3_)Py)_2_]K [71], [PhB(6‐(CF_3_)Py)_3_]K [69], [Cu(CH_3_CN)_4_]BF_4_ [112], and [CuBr(PPh_3_)]_4_ [90, 91] were synthesized as previously reported. All other reactants and reagents were purchased from commercial sources.

#### 4.1.2 Synthesis and Characterization of Metal Complexes

##### 4.1.2.1 [Ph_3_B(3‐(CF_3_)Pz)]Na(THF)_2_ (**1‐Na**)

A solution of 3‐(trifluoromethyl)‐1*H*‐pyrazole (0.136 g, 1 mmol, 1 equiv.) in anhydrous THF (10 mL) was added dropwise to a well‐stirred suspension of dried NaH (0.031 g, 1.1 mmol, 1.1 equiv.) in anhydrous THF (10 mL) at 0°C for 30 min. After filtration through a Celite‐packed frit, BPh_3_ (0.242 g, 1 mmol, 1 equiv.) was added to the solution, and the reaction mixture was stirred at room temperature for 3 h. The solvent was removed under vacuum, giving the ligand as a white solid. Yield: 96% (522 mg, 0.96 mmol). ^1^H NMR (400 MHz, CDCl_3_): δ (ppm) 7.51 (m, 1H, Pz), 7.24 (m, 13H, BPh_3_), 7.15 (m, 2H, BPh_3_), 6.44 (m, 1H, Pz), 3.51 (m, 8H, THF), 1.79 (m, 8H, THF). ^13^C{^1^H} NMR (101 MHz, CDCl_3_): δ (ppm) 155.7 (brs, B‐Ph), 140.6 (q, ^2^ *J*_C‐F_ = 35.2 Hz, *C*‐CF_3_), 138.0 (s, Pz), 134.6 (s, Ph), 127.0 (s, Ph), 124.8 (s, Ph), 123.0 (q, *J*_C‐F_ = 267.3 Hz, *C*F_3_), 101.8 (q, ^3^ *J*_C‐F_ = 1.4 Hz, Pz), 68.1 (THF), 25.4 (THF). ^19^F NMR (376 MHz, CDCl_3_): δ (ppm) −61.83 (s). HR‐MS [ESI, negative ion mode ESI‐TOF]: m/z for C_22_H_17_BF_3_N_2_ [M − Na − 2THF]^−^ calcd: 377.1437. Found: 377.1437.

##### 4.1.2.2 [Cu(PPh_3_){Ph_3_B(3‐(CF_3_)Pz)}] (**7**)

[CuBr(PPh_3_)]_4_ (75 mg, 0.046 mmol, 0.25 equiv.) was added directly to a solution of [Ph_3_B(3‐(CF_3_)Pz)]Na(THF)_2_ (**1‐Na**) (100 mg, 0.184 mmol, 1 equiv.) in anhydrous dichloromethane (20 mL). The reaction mixture was stirred overnight at room temperature and then filtered through a Celite‐packed frit. All volatiles were removed under vacuum to obtain a white powder. The compound was recrystallized in minimum amount of dichloromethane stored at −20°C to obtain high‐quality single crystals. Yield: 90% (162 mg, 0.166 mmol). ^1^H NMR (400 MHz, CDCl_3_): δ (ppm) 7.59 (brs, 1H, Pz), 7.44 (m, 3H, BPh_3_), 7.33 (m, 6H, BPh_3_), 7.25‐7.23 (m, 6H, BPh_3_), 7.06 (m, 9H, PPh_3_), 6.94 (m, 6H, PPh_3_), 6.60 (d, *J* = 1.8 Hz, 1H, Pz). ^13^C{^1^H} NMR (CDCl_3_, 126 MHz): δ (ppm) 150.5 (brs, B‐Ph), 140.2 (q, ^2^ *J*_C‐F_ = 37.1 Hz, *C*‐CF_3_), 137.4 (s, Pz), 134.5 (s, Ph), 133.8 (d, *J*_C‐P_ = 14.9 Hz, PPh_3_), 130.6 (d, *J*_C‐P_ = 1.9 Hz, PPh_3_), 130.3 (d, *J*_C‐P_ = 42.9 Hz, PPh_3_), 128.8 (d, *J*_C‐P_ = 10.1 Hz, PPh_3_), 127.5 (s, Ph), 125.9 (s, Ph), 121.3 (q, *J*_C‐F_ = 268.8 Hz, *C*F_3_), 103.9 (q, ^3^ *J*_C‐F_ = 1.9 Hz, Pz). ^19^F NMR (376 MHz, CDCl_3_): δ (ppm) −60.07 (s). ^31^P{^1^H} (202 MHz, CDCl_3_): δ (ppm) 8.94 (s). ^11^B{^1^H} NMR (128 MHz, CDCl_3_): δ (ppm) −0.40. Anal. calcd for C_40_H_32_BCuF_3_N_2_P: C, 68.34; H, 4.59; N, 3.98%. Found: C, 68.22; H, 4.39; N, 4.06%.

##### 4.1.2.3 [Cu(PPh_3_){Ph_2_B(3‐(CF_3_)Pz)_2_}] (**8**)

To mixture containing [Ph_2_B(3‐(CF_3_)Pz)_2_]Na (100 mg, 0.218 mmol, 1 equiv.) and [Cu(CH_3_CN)_4_]BF_4_ (69 mg, 0.218 mmol, 1 equiv.) was added anhydrous dichloromethane (20 mL). The solution was stirred for 3 h before filtered through a Celite‐packed frit. Solid PPh_3_ (58 mg, 0.218 mmol, 1 equiv.) was added directly to the solution and the mixture was stirred for additional 30 min. All volatiles were removed under vacuum to obtain a white powder. The compound was recrystallized in minimum amount of dichloromethane, layered with hexane and stored at −20°C to obtain high‐quality single crystals. Yield: 91% (151 mg, 0.198 mmol). ^1^H NMR (400 MHz, CDCl_3_): δ (ppm) 7.63 (d, *J* = 1.2 Hz, 2H), 7.46‐7.36 (m, 5H, Ph/PPh_3_), 7.34‐7.30 (m, 10H, Ph/PPh_3_), 7.23‐7.17 (m, 2H, Ph/PPh_3_), 7.17‐7.08 (m, 7H, Ph/PPh_3_), 7.04 (m, 1H, Ph/PPh_3_), 6.51 (d, *J* = 2.3 Hz, 2H). ^13^C{^1^H} NMR (101 MHz, CDCl_3_): δ (ppm) 142.7 (d, ^2^ *J*_F‐C_ = 37.1 Hz, *C*‐CF_3_), 137.8 (s, Pz), 134.5 (s, Ph), 133.9 (d, *J* = 14.9 Hz, PPh_3_), 131.8 (d, *J* = 40.1 Hz, PPh_3_), 130.0 (d, *J* = 2.0 Hz, PPh_3_), 128.4 (d, *J* = 10.1 Hz, PPh_3_), 127.6 (s, Ph), 127.5 (s, Ph), 121.1 (d, ^1^ *J*_F‐C_ = 269.2 Hz, *C*F_3_), 103.3 (q, *J* = 2.1 Hz, Pz). ^19^F NMR (376 MHz, CDCl_3_): δ (ppm) −59.91 (d, *J* = 4.9 Hz). ^31^P{^1^H} NMR (162 MHz, CDCl_3_): δ (ppm) 5.92 (br s). ^11^B{^1^H} NMR (128 MHz, CDCl_3_): δ (ppm) −1.70 (s). HR‐MS [ESI, positive ion mode ESI‐TOF]: m/z for C_32_H_24_BCuF_6_N_2_P [M − Ph]^+^ calcd: 683.1032. Found: 683.1032.

##### 4.1.2.4 [Cu(PPh_3_){PhB(3‐(CF_3_)Pz)_3_}] (**9**)

To mixture containing [PhB(3‐(CF_3_)Pz)_3_]K (100 mg, 0.194 mmol, 1 equiv.) and [Cu(CH_3_CN)_4_]BF_4_ (61 mg, 0.194 mmol, 1 equiv.) was added anhydrous dichloromethane (20 mL). The solution was stirred at room temperature for 3 h and then filtered through a Celite‐packed frit. Solid PPh_3_ (51 mg, 0.194 mmol, 1 equiv.) was added directly to the solution and the mixture was stirred for 30 min. All volatiles were removed under vacuum to obtain a white powder. The compound was recrystallized in minimum amount of dichloromethane, layered with hexane and stored at −20°C to obtain high‐quality single crystals. Yield: 93% (96 mg, 0.180 mmol). ^1^H NMR (400 MHz, CDCl_3_): δ (ppm) 7.50 (s, 3H, Pz), 7.43‐7.37 (m, 3H, Ph/PPh_3_), 7.32 (t, *J* = 6.9 Hz, 6H, PPh_3_), 7.17‐7.01 (m, 7H, Ph/PPh_3_), 6.83 (s, 4H, Ph/PPh_3_), 6.54 (s, 3H, Pz). ^13^C{^1^H} NMR (101 MHz, CDCl_3_): δ (ppm) 143.1 (d, ^2^ *J*_F‐C_ = 44.3 Hz, *C*‐CF_3_), 138.4 (s, Ph), 136.8 (s, Pz), 133.8 (d, *J* = 14.9 Hz, PPh_3_), 133.2 (s, Ph), 131.4 (d, *J* = 40.9 Hz, PPh_3_), 130.2 (d, *J* = 1.4 Hz, PPh_3_), 128.6 (d, *J* = 10.2 Hz, PPh_3_), 128.1 (s, Ph), 120.9 (d, ^1^ *J*_F‐C_ = 270.8 Hz, CF_3_), 104.3 (s, Pz). ^19^F NMR (376 MHz, CDCl_3_): δ (ppm) −60.18 (d, *J* = 1.7 Hz, 6F), −61.40 (brs, 3F). ^31^P{^1^H} (162 MHz, CDCl_3_): δ (ppm) 5.89 (br s). ^11^B{^1^H} NMR (128 MHz, CDCl_3_): δ (ppm) 1.32. HR‐MS [ESI, positive ion mode ESI‐TOF]: m/z for C_36_H_27_BCuF_9_N_6_P [M + H]^+^ calcd: 819.1280. Found: 819.1282.

##### 4.1.2.5 [Cu(PPh_3_){Ph_3_B(6‐(CF_3_)Py)}] (**10**)

To a solution of 6‐(CF_3_)‐2‐PyMgCl(THF)_1.5_ (100 mg, 0.318 mmol, 1 equiv.) in anhydrous THF (20 mL), a solution of BPh_3_ (77 mg, 0.318 mmol, 1 equiv.) in anhydrous THF (5 mL) was added slowly at room temperature. The reaction mixture was stirred for 3 h, after which solid [CuBr(PPh_3_)]_4_ (129.2 mg, 0.0795 mmol, 0.25 equiv.) was added. The combined mixture was stirred overnight, then filtered through a Celite‐packed frit. Volatiles were removed under vacuum, yielding a white powder. The compound was recrystallized in minimum amount of dichloromethane, layered with hexane and stored at −20°C to obtain quality single crystals. Yield: 56% (127 mg, 0.178 mmol). ^1^H NMR (500 MHz, CDCl_3_): δ (ppm) 7.81 (d, *J* = 7.8 Hz, 1H, Py), 7.71 (t, *J* = 7.8 Hz, 1H, Py), 7.53 (d, *J* = 7.7 Hz, 1H, Py), 7.41 (t, *J* = 7.4 Hz, 3H, PPh_3_), 7.31 (t, *J* = 7.6 Hz, 6H, PPh_3_), 7.26 (m, 6H, PPh_3_), 7.00 (m, 9H, BPh), 6.83 (m, 6H, BPh). ^13^C{^1^H} NMR (126 MHz, CDCl_3_): δ (ppm) 192.8 (q, *J* = 42.7 Hz, BPh), 153.7 (q, *J* = 57.2 Hz, BPy), 144.1 (q, ^2^ *J*_C‐F_ = 31.1 Hz, *C*‐CF_3_), 136.4 (s, Py), 136.0 (s, Ph), 134.7 (s, Py), 133.9 (d, *J* = 14.6 Hz, PPh_3_), 130.8 (d, *J* = 40.7 Hz, PPh_3_), 130.3 (d, *J* = 1.5 Hz, PPh_3_), 128.5 (d, *J* = 10.1 Hz, PPh_3_), 127.2 (s, Ph), 125.1 (s, Ph), 121.9 (q, ^1^ *J*_C‐F_ = 274.1 Hz), 116.9 (s, Py). ^19^F NMR (471 MHz, CDCl_3_): δ (ppm) −65.81 (d, *J*_P‐F_ = 10.2 Hz, CF_3_). ^31^P{^1^H} (202 MHz, CDCl_3_): δ (ppm) 6.70 (br s). ^11^B{^1^H} NMR (160 MHz, CDCl_3_): δ (ppm) −7.13 (s). HR‐MS [ESI, positive ion mode ESI‐TOF]: m/z for C_36_H_28_BCuNPF_3_ [M − Ph]^+^ calcd: 636.1300. Found: 636.1284.

##### 4.1.2.6 [Cu(PPh_3_){Ph_2_B(6‐(CF_3_)Py)_2_}] (**11**)

To a mixture of [Ph_2_B(6‐(CF_3_)Py)_2_]K (100 mg, 0.201 mmol, 1 equiv.) and [Cu(CH_3_CN)_4_]BF_4_ (64 mg, 0.201 mmol, 1 equiv.), 20 mL of anhydrous dichloromethane were added. The mixture was allowed to stir for 3 h at room temperature and then filtered through a Celite‐packed frit. Solid PPh_3_ (53 mg, 0.201 mmol, 1 equiv.) was added directly to the filtrate, and the mixture was stirred for additional 30 min. All volatiles were removed under vacuum to obtain a white powder. The compound was recrystallized in minimum amount of dichloromethane, layered with hexane and stored at −20°C to obtain high‐quality single crystals. Yield: 90% (142 mg, 0.181 mmol). ^1^H NMR (400 MHz, CDCl_3_): δ (ppm) 7.72 (d, *J* = 7.7 Hz, 2H, Py), 7.63 (t, *J* = 7.7 Hz, 2H, Py), 7.48 (d, *J* = 7.8 Hz, 2H, Py), 7.35 (t, *J* = 7.2 Hz, 4H, Ph/PPh_3_), 7.28‐7.09 (m, 8H, Ph/PPh_3_), 6.95‐6.82 (m, 8H, Ph/PPh_3_), 6.65 (s, 2H, Ph/PPh_3_), 6.38 (s, 3H, Ph/PPh_3_). ^13^C{^1^H} NMR (101 MHz, CDCl_3_): δ (ppm) 188.3 (q, ^1^ *J*_C‐B_ = 56.6 Hz, B‐Py), 145.3 (d, ^2^ *J*_C‐F_ = 36.1 Hz, *C*‐CF_3_), 136.5 (s, Py), 135.9 (s, Py), 135.3, 134.8 (s, Ph), 134.0 (d, *J* = 14.1 Hz, PPh_3_), 132.1 (d, *J* = 38.3 Hz, PPh_3_), 129.8 (d, *J* = 1.9 Hz, PPh_3_), 128.2 (d, *J* = 9.8 Hz, PPh_3_), 127.0 (s, Ph), 124.5 (s, Ph), 122.2 (d, ^1^ *J*_C‐F_ = 274.5 Hz, *C*F_3_), 117.3 (s, Py). ^19^F NMR (376 MHz, CDCl_3_): δ (ppm) −65.16 (d, *J*_F‐P_ = 10.7 Hz). ^31^P{^1^H} (162 MHz, CDCl_3_): δ (ppm) 4.06 (br s). ^11^B{^1^H} NMR (128 MHz, CDCl_3_): δ (ppm) −7.75. HR‐MS [ESI, positive ion mode ESI‐TOF]: m/z for C_42_H_32_BCuF_6_N_2_P [M + H]^+^ calcd: 783.1596. Found: 783.1599.

##### 4.1.2.7 [Cu(PPh_3_){PhB(6‐(CF_3_)Py)_3_}] (**12**)

To mixture containing [PhB(6‐(CF_3_)Py)_3_]K (100 mg, 0.194 mmol, 1 equiv.) and [Cu(CH_3_CN)_4_]BF_4_ (61 mg, 0.194 mmol, 1 equiv.) was added anhydrous dichloromethane (20 mL). The solution was stirred for 3 h and then filtered through a Celite‐packed frit. Solid PPh_3_ (51 mg, 0.194 mmol, 1 equiv.) was added directly to the solution and the mixture was stirred for additional 30 min. All volatiles were removed under vacuum to obtain a white powder. The compound was recrystallized in minimum amount of dichloromethane, layered with hexane and stored at −20°C to obtain high‐quality single crystals. Yield: 93% (96 mg, 0.180 mmol). ^1^H NMR (400 MHz, CD_2_Cl_2_, −20°C): δ (ppm) 7.66 (t, *J* = 7.9 Hz, 2H), 7.53‐7.50 (m, 5H), 7.42 (d, *J* = 7.7 Hz, 1H), 7.38 (t, *J* = 6.8 Hz, 3H), 7.25 (m, 8H), 7.23‐6.73 (br, 4H), 6.73 (brs, 2H), 6.63 (d, *J* = 7.6 Hz, 1H), 6.36‐6.35 (m, 3H). ^13^C{^1^H} NMR (126 MHz, CD_2_Cl_2,_ −20°C): δ (ppm) 146.5 (q, ^2^ *J*_C‐F_ = 34.0 Hz, *C*‐CF_3_, Py), 145.4 (q, ^2^ *J*_C‐F_ = 34.8 Hz, *C*‐CF_3_, Py), 136.3 (s, Ph), 135.4 (s, Py), 135.2 (s, Py), 134.6 (s, Py), 134.3 (s, Py), 133.8 (d, *J* = 14.1 Hz, PPh_3_), 133.2 (s, PPh_3_), 130.0 (s, PPh_3_), 128.4 (d, *J* = 9.7 Hz, PPh_3_), 127.6 (s, Ph), 125.4 (s, Ph), 122.8 (q, ^1^ *J*_C‐F_ = 274.1, Hz, *C*F_3_), 122.3 (q, ^1^ *J*_C‐F_ = 274.5, Hz, *C*F_3_), 117.9 (s, Py), 115.9 (s, Py). ^19^F NMR (471 MHz, CD_2_Cl_2_, −20°C): δ (ppm) −65.2 (d, *J* = 10.8 Hz, 6F, CF_3_), −67.6 (s, 3F, CF_3_). ^31^P{^1^H} (202 MHz, CD_2_Cl_2_, −20°C): δ (ppm) 3.37 (br). ^11^B{^1^H} NMR (160 MHz, CD_2_Cl_2_, −20°C): δ (ppm) −7.83 (s). HR‐MS [ESI, positive ion mode ESI‐TOF]: m/z for C_42_H_30_BCuF_9_N_3J_P [M + H]^+^ calcd: 852.1423. Found: 852.1429.

### 4.2 X‐Ray Structure Determinations

A suitable crystal covered with a layer of hydrocarbon/Paratone‐N oil was selected and mounted on a Cryo‐loop, and immediately placed in the low temperature nitrogen stream. The X‐ray intensity data of compounds **7–10** and **12** were measured at 100(2) K and the compound **11** was collected at 299(2) K (due to crystal cracking issues at low temperatures) on a Bruker D8 Quest equipped with a PHOTON II 7 CPAD detector and an Oxford Cryosystems 700 series cooler, a Triumph monochromator, and a Mo Kα fine‐focus sealed tube (*λ* = 0.71073 Å). Intensity data were processed using the Bruker Apex program suite. Absorption corrections were applied by using SADABS [113]. Initial atomic positions were located by SHELXT [114], and the structures of the compounds were refined by the least‐squares method using SHELXL [115] within Olex2 GUI [116]. All the non‐hydrogen atoms were refined anisotropically. Hydrogen atoms were included at calculated positions and refined using appropriate riding models. X‐ray structural figures were generated using Olex2. CCDC 2470669–2470674 files contain the supplementary crystallographic data. These data can be obtained free of charge via http://www.ccdc.cam.ac.uk/conts/retrieving.html or from the Cambridge Crystallographic Data Centre (CCDC), 12 Union Road, Cambridge, CB2 1EZ, UK).

### 4.3 Cyclic Voltammetry

Cyclic voltammetry measurements were conducted using a three‐electrode setup in a closed‐compartment cell using a CHI 604B electrochemical analyzer potentiostat. Glassy‐carbon electrode (geometrical area = 3.1 sq mm) was used as the working electrode. A non‐aqueous Ag/Ag^+^ reference electrode was employed, while a Pt wire acted as the counter electrode. The working electrode surface was polished with 0.3 µm alumina powder slurry on a wet microcloth and then washed in acetone and dried under N_2_ prior to use. To maintain an inert environment, N_2_ gas blanket was maintained over the solution during the experiments. Ferrocene was added as an internal standard in all the experiments. All potentials in this study are referenced with respect to ferrocene. Acetonitrile was used as the solvent, and the supporting electrolyte was tetrabutylammonium hexafluorophosphate (TBAPF_6_). The solvents and electrolytes were purchased from commercial sources. The solvent was dried using a solvent purification system, then degassed using the freeze‐pump‐thaw technique. The concentration of the metal complexes and ligand in each case was ∼2 mM.

### 4.4 Experiments with Cultured Human Cancer Cells

Copper complexes were dissolved in DMSO immediately before use, and a calculated amount of drug solution was added to the cell growth medium to a final DMSO concentration of 0.5%, which had no detectable effects on cell viability. Cisplatin was dissolved in 0.9% sodium chloride solution. MTT (3‐(4,5‐dimethylthiazol‐2‐yl)‐2,5‐diphenyltetrazolium bromide) and cisplatin were obtained from Sigma Chemical Co, St. Louis, MO, USA.

#### 4.4.1 Cell Cultures

Human colon (HCT‐15), non‐small cell lung (A549), breast (MCF‐7), and pancreatic (PSN‐1) carcinoma cell lines along with human embryonal teratocarcinoma cells (NTERA‐2) were obtained by American Type Culture Collection (ATCC, Rockville, MD, USA). Human ovarian 2008 cancer cells and their cisplatin‐resistant subline, C13* cells, were kindly provided by Prof. G. Marverti (Dept. of Biomedical Science of Modena University, Italy). Cell lines were maintained in the logarithmic phase at 37°C in a 5% carbon dioxide atmosphere using RPMI‐1640 (HCT‐15, MCF‐7, PSN‐1, NTERA‐2, 2008 and C13*) or F‐12 HAMs (A549) medium (Euroclone) containing 10% fetal calf serum (EuroClone, Milan, Italy), antibiotics (50 units/mL penicillin and 50 µg/mL streptomycin) and 2 mM l‐glutamine.

#### 4.4.2 MTT Assay

The growth inhibitory effect toward tumor cells was evaluated by means of MTT assay as previously described [117]. The IC_50_ values, corresponding to the drug concentrations that reduce the mean absorbance at 570 nm to 50% of that in untreated control wells, were calculated by the four‐parameter logistic (4‐PL) model.

#### 4.4.3 Spheroid Cultures and Acid Phosphatase (APH) Assay

Spheroid cultures were obtained by seeding 2.5 × 10^3^ HCT‐15 cells/well in a round‐bottom non‐treated tissue culture 96‐well plate (Greiner Bio‐one, Kremsmünster, Austria) in phenol red free RPMI media (Sigma Chemical Co., St. Louis, MO, USA) containing 10% fetal calf serum and supplemented with 20% methyl cellulose stock solution. APH modified assay was employed for evaluating cell viability in 3D spheroids. IC_50_ values (drug concentrations that reduce the mean absorbance at 405 nm to 50% of that in untreated control wells) were calculated by 4‐PL model.

#### 4.4.4 Quantification of Thiols

HCT‐15 cells (1.5 × 10^5^) were seeded in a six‐well plate in growth medium (4 mL). After 24 h, cells were incubated for 24 h with tested compounds. Subsequently, the thiol content was measured as previously described [118].

#### 4.4.5 Hydrogen peroxide and Hydroxyl radical detection

The cellular levels of H_2_O_2_ or **^∙^** OH were measured in HCT‐15 cells (10^4^ per well) grown for 24 h in a 96‐well plate in RPMI medium without phenol red (Sigma Chemical Co.). Cells were then washed with PBS and loaded with 10 µM 5‐(and‐6)‐ chloromethyl‐2′,7′‐dichlorodihydrofluorescein diacetate acetyl ester (CM–H_2_DCFDA) (Molecular Probes‐Invitrogen, Eugene, OR) or RH‐EDA (MedChemexpress MCE) for 25 min, in the dark. Afterwards, cells were washed with PBS and incubated with increasing concentrations of tested compounds. Fluorescence increase was estimated utilizing the wavelengths of 485 nm (excitation) and 527 nm (emission) for H_2_O_2_ and 546 nm (excitation) and 566 nm (emission) for **^∙^** OH with an Infinite 200 PRO (Tecan, Switzerland) plate reader.

### 4.5 Protein Disulfide Isomerase (PDI) Activity

The reductase activity of PDI was assayed by measuring the PDI‐catalyzed reduction of insulin in the presence of increasing concentrations of the tested compounds by using PROTEOSTAT PDI assay kit (Enzo Life Sciences, Lausen, Switzerland). Experiments were performed according to the manufacturer's instructions. Briefly, copper complexes or bacitracin (at increasing concentrations) were added to an insulin PDI solution. Subsequently, DTT was added to start PDI reduction activity and after 30 min of incubation, the reaction was stopped by adding the stop reagent mixture. The insulin precipitate was labelled with the fluorescent Proteostat PDI detection reagent and fluorescence intensity was measured at 500 nm excitation and 603 nm emission. IC_50_ values were calculated by 4‐PL model.

### 4.6 Confocal Microscopy Morphological Analyses

HCT‐15 cells were seeded into 8‐well tissue‐culture slides (BD Falcon, Bedford, MA, USA) at 5 × 10^4^ cells/well (0.8 cm^2^). After 24 h, the cells were washed twice with PBS, and following 24 h of treatment with IC_50_ doses of the tested compound, cells were stained for 5 min with 10 µg/mL of Hoechst 33342 (Sigma–Aldrich, St. Louis, MI, USA) in PBS. Samples were examined at 10 × magnification in a Zeiss LSM 800 confocal microscope using the Zeiss ZEN 2.3 software system.

### 4.7 Transmission Electron Microscopy (TEM) Analyses

About 10^6^ HCT‐15 cells were seeded in 24‐well plates and, after 24 h incubation, were treated with IC_50_ concentrations of tested compounds and incubated for additional 24 h. Cells were then washed with cold PBS, harvested and directly fixed with 1.5% glutaraldehyde buffer with 0.2 M sodium cacodylate, pH 7.4. After washing with buffer and postfixation with 1% OsO_4_ in 0.2 M cacodylate buffer, specimens were dehydrated and embedded in epoxy resin (Epon Araldite). Sagittal serial sections (1 µm) were counterstained with toluidine blue; thin sections (90 nm) were given contrast by staining with uranyl acetate and lead citrate. Micrographs were taken with a Hitachi H‐600 electron microscope (Hitachi, Tokyo, Japan) operating at 75 kV. All photos were typeset in Corel Draw 11.

### 4.8 Statistical Analysis

All values are the means ± SD of no less than three measurements starting from three different cell cultures. Multiple comparisons were made by ANOVA followed by the Tukey–Kramer multiple comparison test (*p *<* 0.05, **p *<* 0.01), using GraphPad software.

## Conflicts of Interest

The authors declare no conflicts of interest.

## Supporting information



Example of different coordination modes (Figure S1), multinuclear NMR spectra (Figures S2–S37), cyclic voltammograms (Figures S38–S41), selected structural and spectroscopic parameters (Table S1) and X‐ray crystal parameters (Tables S2–S7) are provided in the Supporting Information.
The authors have cited additional references within the Supporting Information.

## Data Availability

The data that supports the findings of this study are available in the supplementary material of this article.
