# Supplementary material for: Structural and Biological Insights Into Copper(I) Phosphane Complexes With Boron‐Phenylated Poly(3‐(CF3)Pyrazolyl)‐ and Poly(6‐(CF3)‐2‐pyridyl)‐Borates
Source: Chemistry. 2026 Mar 4;32(19):e70848. doi: 10.1002/chem.70848 (PMC13206215; doi:10.1002/chem.70848)
Supplement: Supplementary file 1 — Example of different coordination modes (Figure S1), multinuclear NMR spectra (Figures S2–S37), cyclic voltammograms (Figures S38–S41), selected structural and spectroscopic parameters (Table S1) and X‐ray crystal parameters (Tables S2–S7) are provided in the Supporting Information. The authors have cited additional references within the Supporting Information. [file CHEM-32-e70848-s001.docx]

**Supporting Information**

**Structural and biological insights into copper(I) phosphane complexes with boron-phenylated poly(3-(CF_3_)pyrazolyl)- and poly(6-(CF_3_)-2-pyridyl)-borates**

Vo Quang Huy Phan,^[a],†^ Jo’ Del Gobbo,^b,†^ Mukundam Vanga,^[a]^ Deepika V. Karade, ^[a]^ Cristina Marzano,^[c]^ Valentina Gandin,*^[c]^ Laura Rigon,^[d]^ Carlo Santini,^[b]^ Maura Pellei,*^[b]^ H. V. Rasika Dias*^[a]^

^†^ contributed equally to this work

[a] *Department of Chemistry and Biochemistry, P.O. Box 19065, The University of Texas at Arlington, Arlington, Texas 76019-0065, USA.*

[b] *School of Science and Technology, Chemistry Division, University of Camerino, via Madonna delle Carceri (ChIP), 62032 Camerino, Italy.*

[c] *Department of Pharmaceutical and Pharmacological Sciences, University of Padova, Via Marzolo 5, 35131 Padova, Italy.*

[d] *Department of Medicine, University of Padova, Via Giustiniani 2, 35128 Padova, Italy.*

**Table of Contents**

**Figure S1**. Example of different coordination modes illustrated using a *B*-phenylated tris(pyrazolyl)borate ligand to metal sites

**1. Spectroscopic Data**

**Figure S2**. ^1^H NMR of [Ph_3_B(3-(CF_3_)Pz)]Na(THF)_2_ (**1-Na**) in CDCl_3_

**Figure S3**. ^13^C{^1^H} NMR of [Ph_3_B(3-(CF_3_)Pz)]Na(THF)_2_ (**1-Na**) in CDCl_3_

**Figure S5**. ^1^H NMR of [Cu(PPh_3_){Ph_3_B(3-(CF_3_)Pz)}] (**7**) in CDCl_3_

**Figure S6**. ^13^C{^1^H} NMR of [Cu(PPh_3_){Ph_3_B(3-(CF_3_)Pz)}] (**7**) in CDCl_3_

**Figure S7**. ^19^F NMR of [Cu(PPh_3_){Ph_3_B(3-(CF_3_)Pz)}] (**7**) in CDCl_3_

**Figure S8**. ^31^P{^1^H} NMR of [Cu(PPh_3_){Ph_3_B(3-(CF_3_)Pz)}] (**7**) in CDCl_3_

**Figure S9**. ^11^B{^1^H} NMR of [Cu(PPh_3_){Ph_3_B(3-(CF_3_)Pz)}] (**7**) in CDCl_3_

**Figure S10**. ^1^H NMR of [Cu(PPh_3_){Ph_2_B(3-(CF_3_)Pz)_2_}] (**8**) in CDCl_3_

**Figure S11**. ^13^C{^1^H} NMR of [Cu(PPh_3_){Ph_2_B(3-(CF_3_)Pz)_2_}] (**8**) in CDCl_3_

**Figure S12**. ^19^F NMR of [Cu(PPh_3_){Ph_2_B(3-(CF_3_)Pz)_2_}] (**8**) in CDCl_3_

**Figure S13**. ^31^P{^1^H} NMR of [Cu(PPh_3_){Ph_2_B(3-(CF_3_)Pz)_2_}] (**8**) in CDCl_3_

**Figure S14**. ^11^B{^1^H} NMR of [Cu(PPh_3_){Ph_2_B(3-(CF_3_)Pz)_2_}] (**8**) in CDCl_3_

**Figure S15**. ^1^H NMR of [Cu(PPh_3_){PhB(3-(CF_3_)Pz)_3_}] (**9**) in CDCl_3_

**Figure S16**. ^13^C{^1^H} NMR of [Cu(PPh_3_){PhB(3-(CF_3_)Pz)_3_}] (**9**) in CDCl_3_

**Figure S17**. ^19^F NMR of [Cu(PPh_3_){PhB(3-(CF_3_)Pz)_3_}] (**9**) in CDCl_3_

**Figure S18**. ^31^P{^1^H} NMR of [Cu(PPh_3_){PhB(3-(CF_3_)Pz)_3_}] (**9**) in CDCl_3_

**Figure S19**. ^11^B{^1^H} NMR of [Cu(PPh_3_){PhB(3-(CF_3_)Pz)_3_}] (**9**) in CDCl_3_

**Figure S20**. ^1^H NMR of [Cu(PPh_3_){Ph_3_B(6-(CF_3_)Py)}] (**10**) in CDCl_3_

**Figure S21**. ^13^C{^1^H} NMR of [Cu(PPh_3_){Ph_3_B(6-(CF_3_)Py)}] (**10**) in CDCl_3_

**Figure S22**. ^19^F NMR of [Cu(PPh_3_){Ph_3_B(6-(CF_3_)Py)}] (**10**) in CDCl_3_

**Figure S23**. ^31^P{^1^H} NMR of [Cu(PPh_3_){Ph_3_B(6-(CF_3_)Py)}] (**10**) in CDCl_3_

**Figure S24**. ^11^B{^1^H} NMR of [Cu(PPh_3_){Ph_3_B(6-(CF_3_)Py)}] (**10**) in CDCl_3_

**Figure S25**. ^1^H NMR of [Cu(PPh_3_){Ph_2_B(6-(CF_3_)Py)_2_}] (**11**) in CDCl_3_

**Figure S26**. ^13^C{^1^H} NMR of [Cu(PPh_3_){Ph_2_B(6-(CF_3_)Py)_2_}] (**11**) in CDCl_3_

**Figure S27**. ^19^F NMR of [Cu(PPh_3_){Ph_2_B(6-(CF_3_)Py)_2_}] (**11**) in CDCl_3_

**Figure S28**. ^31^P{^1^H} NMR of [Cu(PPh_3_){Ph_2_B(6-(CF_3_)Py)_2_}] (**11**) in CDCl_3_

**Figure S29**. ^11^B{^1^H} NMR of [Cu(PPh_3_){Ph_2_B(6-(CF_3_)Py)_2_}] (**11**) in CDCl_3_

**Figure S30**. ^1^H NMR of [Cu(PPh_3_){PhB(6-(CF_3_)Py)_3_}] (**12**) in CDCl_3_

**Figure S31**. ^19^F NMR of [Cu(PPh_3_){PhB(6-(CF_3_)Py)_3_}] (**12**) in CDCl_3_

**Figure S32**. ^1^H NMR of [Cu(PPh_3_){PhB(6-(CF_3_)Py)_3_}] (**12**) in CD_2_Cl_2_ at -20 °C

**Figure S33**. ^19^F NMR of [Cu(PPh_3_){PhB(6-(CF_3_)Py)_3_}] (**12**) in CD_2_Cl_2_ at -20 °C

**Figure S34**. ^19^F NMR of [Cu(PPh_3_){PhB(6-(CF_3_)Py)_3_}] (**12**) in CD_2_Cl_2_ at 20 °C and -20 °C

**Figure S35**. ^13^C{^1^H} NMR of [Cu(PPh_3_){PhB(6-(CF_3_)Py)_3_}] (**12**) in CD_2_Cl_2_ at -20 °C

**Figure S36**. ^31^P{^1^H} NMR of [Cu(PPh_3_){PhB(6-(CF_3_)Py)_3_}] (**12**) in CD_2_Cl_2_ at -20 °C

**Figure S37**. ^11^B{^1^H} NMR of [Cu(PPh_3_){PhB(6-(CF_3_)Py)_3_}] (**12**) in CD_2_Cl_2_ at -20 °C

**Table S1**. Selected structural and spectroscopic parameters (at room temperature) of fluorinated tris(pyridyl)borate and tris(pyrazolyl)borate copper(I) triphenylphosphane complexes.

**2. X-ray Crystallographic Data**

**X-ray structure determinations**

**Table S2**. Crystal data and structure refinement for [Cu(PPh_3_){Ph_3_B(3-(CF_3_)Pz)}] (**7**)

**Table S3**. Crystal data and structure refinement for [Cu(PPh_3_){Ph_2_B(3-(CF_3_)Pz)_2_}] (**8**)

**Table S4**. Crystal data and structure refinement for [Cu(PPh_3_){PhB(3-(CF_3_)Pz)_3_}] (**9**)

**Table S5.** Crystal data and structure refinement for [Cu(PPh_3_){Ph_3_B(6-(CF_3_)Py)}] (**10**)

**Table S6**. Crystal data and structure refinement for [Cu(PPh_3_){Ph_2_B(6-(CF_3_)Py)_2_}] (**11**)

**Table S7**. Crystal data and structure refinement for [Cu(PPh_3_){PhB(6-(CF_3_)Py)_3_}] (**12**)

**3. Electrochemistry**

**Figure S38.** Cyclic voltammograms of 2 mM solution of complexes (a) [Cu(PPh_3_){Ph_3_B(3-(CF_3_)Pz)}] (**7**), (b) [Cu(PPh_3_){Ph_2_B(3-(CF_3_)Pz)_2_}] (**8**), and (c) [Cu(PPh_3_){PhB(3-(CF_3_)Pz)_3_}] (**9**) in 0.1 M TBAPF_6_/ACN under N_2_ atmosphere.

**Figure S39.** Cyclic voltammograms of 2 mM solution of complexes (a) [Ph_3_B(3-(CF_3_)Pz)]Na(THF)_2_, (b) [Ph_2_B(3-(CF_3_)Pz)_2_]Na, and (c) [PhB(3-(CF_3_)Pz)_3_]K in 0.1 M TBAPF_6_/ACN under N_2_ atmosphere.

**Figure S40.** Cyclic voltammograms of 2 mM solution of complexes (a) [Cu(PPh_3_){Ph_3_B(6-(CF_3_)Py)}] (**10**), (b) [Cu(PPh_3_){Ph_2_B(6-(CF_3_)_2_Py)_2_}] (**11**), and (c) [Cu(PPh_3_){PhB(6-(CF_3_)_2_Py)_3_}] (**12**) in 0.1 M TBAPF_6_/ACN under N_2_ atmosphere.

**Figure S41.** Cyclic voltammograms of 2 mM solution of complexes (a) [Ph_2_B(6-(CF_3_)_2_Py)_2_]H, and (b) [PhB(6-(CF_3_)_2_Py)_3_]H in 0.1 M TBAPF_6_/ACN under N_2_ atmosphere.

**4. References**

**Figure S1**. Example of different coordination modes illustrated using a *B*-phenylated tris(pyrazolyl)borate ligand to metal sites

**1. Spectroscopic Data**

**Figure S2**. ^1^H NMR of [Ph_3_B(3-(CF_3_)Pz)]Na(THF)_2_ (**1-Na**) in CDCl_3_

**Figure S3**. ^13^C{^1^H} NMR of [Ph_3_B(3-(CF_3_)Pz)]Na(THF)_2_ (**1-Na**) in CDCl_3_

**Figure S4**. ^19^F NMR of [Ph_3_B(3-(CF_3_)Pz)]Na(THF)_2_ (**1-Na**) in CDCl_3_

**Figure S5**. ^1^H NMR of [Cu(PPh_3_){Ph_3_B(3-(CF_3_)Pz)}] (**7**) in CDCl_3_

**Figure S6**. ^13^C{^1^H} NMR of [Cu(PPh_3_){Ph_3_B(3-(CF_3_)Pz)}] (**7**) in CDCl_3_

**Figure S7**. ^19^F NMR of [Cu(PPh_3_){Ph_3_B(3-(CF_3_)Pz)}] (**7**) in CDCl_3_

**Figure S8**. ^31^P{^1^H} NMR of [Cu(PPh_3_){Ph_3_B(3-(CF_3_)Pz)}] (**7**) in CDCl_3_

**Figure S9**. ^11^B{^1^H} NMR of [Cu(PPh_3_){Ph_3_B(3-(CF_3_)Pz)}] (**7**) in CDCl_3_

**Figure S10**. ^1^H NMR of [Cu(PPh_3_){Ph_2_B(3-(CF_3_)Pz)_2_}] (**8**) in CDCl_3_

**Figure S11**. ^13^C{^1^H} NMR of [Cu(PPh_3_){Ph_2_B(3-(CF_3_)Pz)_2_}] (**8**) in CDCl_3_

**Figure S12**. ^19^F NMR of [Cu(PPh_3_){Ph_2_B(3-(CF_3_)Pz)_2_}] (**8**) in CDCl_3_

**Figure S13**. ^31^P{^1^H} NMR of [Cu(PPh_3_){Ph_2_B(3-(CF_3_)Pz)_2_}] (**8**) in CDCl_3_

**Figure S14**. ^11^B{^1^H} NMR of [Cu(PPh_3_){Ph_2_B(3-(CF_3_)Pz)_2_}] (**8**) in CDCl_3_

**Figure S15**. ^1^H NMR of [Cu(PPh_3_){PhB(3-(CF_3_)Pz)_3_}] (**9**) in CDCl_3_

**Figure S16**. ^13^C{^1^H} NMR of [Cu(PPh_3_){PhB(3-(CF_3_)Pz)_3_}] (**9**) in CDCl_3_

**Figure S17**. ^19^F NMR of [Cu(PPh_3_){PhB(3-(CF_3_)Pz)_3_}] (**9**) in CDCl_3_

**Figure S18**. ^31^P{^1^H} NMR of [Cu(PPh_3_){PhB(3-(CF_3_)Pz)_3_}] (**9**) in CDCl_3_

**Figure S19**. ^11^B{^1^H} NMR of [Cu(PPh_3_){PhB(3-(CF_3_)Pz)_3_}] (**9**) in CDCl_3_

**Figure S20**. ^1^H NMR of [Cu(PPh_3_){Ph_3_B(6-(CF_3_)Py)}] (**10**) in CDCl_3_

**Figure S21**. ^13^C{^1^H} NMR of [Cu(PPh_3_){Ph_3_B(6-(CF_3_)Py)}] (**10**) in CDCl_3_

**Figure S22**. ^19^F NMR of [Cu(PPh_3_){Ph_3_B(6-(CF_3_)Py)}] (**10**) in CDCl_3_

**Figure S23**. ^31^P{^1^H} NMR of [Cu(PPh_3_){Ph_3_B(6-(CF_3_)Py)}] (**10**) in CDCl_3_

**Figure S24**. ^11^B{^1^H} NMR of [Cu(PPh_3_){Ph_3_B(6-(CF_3_)Py)}] (**10**) in CDCl_3_

**Figure S25**. ^1^H NMR of [Cu(PPh_3_){Ph_2_B(6-(CF_3_)Py)_2_}] (**11**) in CDCl_3_

**Figure S26**. ^13^C{^1^H} NMR of [Cu(PPh_3_){Ph_2_B(6-(CF_3_)Py)_2_}] (**11**) in CDCl_3_

**Figure S27**. ^19^F NMR of [Cu(PPh_3_){Ph_2_B(6-(CF_3_)Py)_2_}] (**11**) in CDCl_3_

**Figure S28**. ^31^P{^1^H} NMR of [Cu(PPh_3_){Ph_2_B(6-(CF_3_)Py)_2_}] (**11**) in CDCl_3_

**Figure S29**. ^11^B{^1^H} NMR of [Cu(PPh_3_){Ph_2_B(6-(CF_3_)Py)_2_}] (**11**) in CDCl_3_

**Figure S30**. ^1^H NMR of [Cu(PPh_3_){PhB(6-(CF_3_)Py)_3_}] (**12**) in CDCl_3_

**Figure S31**. ^19^F NMR of [Cu(PPh_3_){PhB(6-(CF_3_)Py)_3_}] (**12**) in CDCl_3_

**Figure S32**. ^1^H NMR of [Cu(PPh_3_){PhB(6-(CF_3_)Py)_3_}] (**12**) in CD_2_Cl_2_ at -20 °C

**Figure S33**. ^19^F NMR of [Cu(PPh_3_){PhB(6-(CF_3_)Py)_3_}] (**12**) in CD_2_Cl_2_ at -20 °C

**Figure S34**. ^19^F NMR of [Cu(PPh_3_){PhB(6-(CF_3_)Py)_3_}] (**12**) in CD_2_Cl_2_
at 20 °C (top) and -20 °C (bottom)

**Figure S35**. ^13^C{^1^H} NMR of [Cu(PPh_3_){PhB(6-(CF_3_)Py)_3_}] (**12**) in CD_2_Cl_2_ at -20 °C

**Figure S36**. ^31^P{^1^H} NMR of [Cu(PPh_3_){PhB(6-(CF_3_)Py)_3_}] (**12**) in CD_2_Cl_2_ at -20 °C

**Figure S37**. ^11^B{^1^H} NMR of [Cu(PPh_3_){PhB(6-(CF_3_)Py)_3_}] (**12**) in CD_2_Cl_2_ at -20 °C

**Table S1**. Selected structural and spectroscopic parameters (at room temperature) of fluorinated tris(pyridyl)borate and tris(pyrazolyl)borate copper(I) triphenylphosphane complexes, [Cu(PPh_3_){Ph_3_B(3-(CF_3_)Pz)}] (**7**), [Cu(PPh_3_){Ph_2_B(3-(CF_3_)Pz)_2_}] (**8**), [Cu(PPh_3_){PhB(3-(CF_3_)Pz)_3_}] (**9**), [Cu(PPh_3_){Ph_3_B(6-(CF_3_)Py)}] (**10**), [Cu(PPh_3_){Ph_2_B(6-(CF_3_)Py)_2_}] (**11**) and [Cu(PPh_3_){PhB(6-(CF_3_)Py)_3_}] (**12**).

| **Compound** | **Solvent** | **^19^F NMR**  δ (ppm) | **Multiplicity**  *J*_P-F_ (Hz) | **P•••*C*F_3_**  Å | **^31^P{^1^H} NMR**  δ (ppm) | **Reference** |
| --- | --- | --- | --- | --- | --- | --- |
| **7** | CDCl_3_ | −60.07 | s | 4.680 | 8.94 | This work |
| **8** | CDCl_3_ | −59.91 | d, 4.9 | 4.481 | 5.92 | This work |
| **9** | CDCl_3_ | −60.18 | d, 1.7 | 4.553 | 5.89 | This work |
| **10** | CDCl_3_ | −65.81 | d, 10.2 | 4.389 | 6.70 | This work |
| **11** | CDCl_3_ | −65.16 | d, 10.7 | 4.157 | 4.06 | This work |
| **12** | CDCl_3_ | −65.6  −66.2 | d, 11.1  d, 5.8 | 4.148 | 3.37 | This work |
| [Cu(PPh_3_){HB(3,5-(CF_3_)_2_Pz)_3_}] | C_6_D_6_ | -59.6 | d, 6.5 | 4.292 | 7.2 | ^[1]^ |
| [Cu(PPh_3_){H_2_B(3,5-(CF_3_)_2_Pz)_2_}] | C_6_D_6_ | -60.5 | s | 4.476 | 3.37 | ^[2]^ |
| [Cu(PPh_3_)_2_{H_2_B(3,5-(CF_3_)_2_Pz)_2_}] | C_6_D_6_ | -60.65 | s | 4.521 | -25.72 | ^[3]^ |
| [Cu(PPh_3_)_2_{H_3_B(5-(CF_3_)Pz)}] | DMSO-d_6_ | -59.40 | s | 6.245 | -0.85 | ^[4]^ |

**2. X-ray Crystallographic Data**


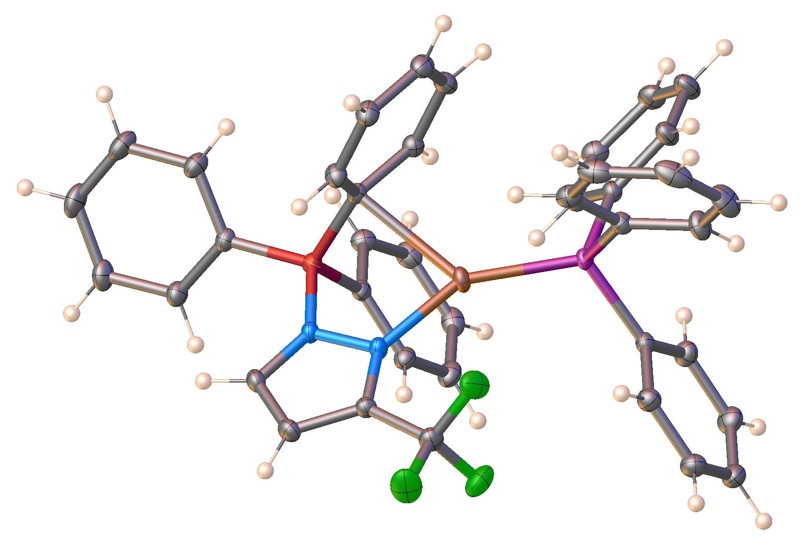


| **Table S2**. Crystal data and structure refinement for [Cu(PPh_3_){Ph_3_B(3-(CF_3_)Pz)}] (**7**) | |
| --- | --- |
| Identification code | HRD194_a |
| Empirical formula | C_40_H_32_BCuF_3_N_2_P |
| Formula weight | 702.99 |
| Temperature/K | 100.00 |
| Crystal system | triclinic |
| Space group | P-1 |
| a/Å | 9.4367(3) |
| b/Å | 12.6998(4) |
| c/Å | 15.1185(4) |
| α/° | 90.0950(10) |
| β/° | 91.5330(10) |
| γ/° | 111.1150(10) |
| Volume/Å^3^ | 1689.48(9) |
| Z | 2 |
| ρ_calc_g/cm^3^ | 1.382 |
| μ/mm^‑1^ | 0.742 |
| F(000) | 724.0 |
| Crystal size/mm^3^ | 0.3 × 0.15 × 0.13 |
| Radiation | Mo Kα (λ = 0.71073) |
| 2Θ range for data collection/° | 5.392 to 66.268 |
| Index ranges | -14 ≤ h ≤ 14, -19 ≤ k ≤ 19, -23 ≤ l ≤ 23 |
| Reflections collected | 33157 |
| Independent reflections | 12677 [R_int_ = 0.0183, R_sigma_ = 0.0218] |
| Data/restraints/parameters | 12677/0/433 |
| Goodness-of-fit on F^2^ | 1.036 |
| Final R indexes [I>=2σ (I)] | R_1_ = 0.0286, wR_2_ = 0.0752 |
| Final R indexes [all data] | R_1_ = 0.0352, wR_2_ = 0.0799 |
| Largest diff. peak/hole / e Å^-3^ | 0.49/-0.62 |


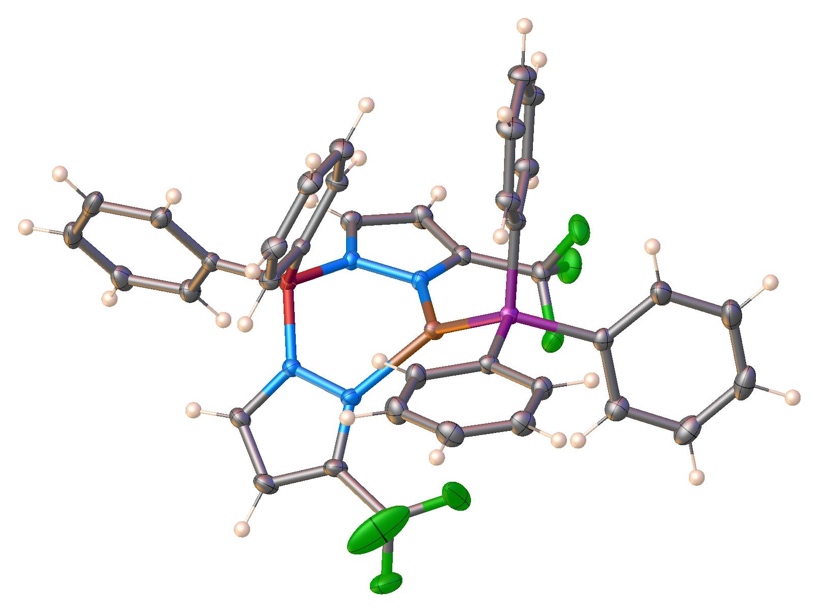


| **Table S3**. Crystal data and structure refinement for [Cu(PPh_3_){Ph_2_B(3-(CF_3_)Pz)_2_}] (**8**) | |
| --- | --- |
| Identification code | HRD243_a |
| Empirical formula | C_38_H_29_BCuF_6_N_4_P |
| Formula weight | 760.97 |
| Temperature/K | 100.00 |
| Crystal system | monoclinic |
| Space group | P2_1_/c |
| a/Å | 20.8496(7) |
| b/Å | 16.1697(5) |
| c/Å | 10.1443(3) |
| α/° | 90 |
| β/° | 90.501(2) |
| γ/° | 90 |
| Volume/Å^3^ | 3419.84(19) |
| Z | 4 |
| ρ_calc_g/cm^3^ | 1.478 |
| μ/mm^‑1^ | 0.753 |
| F(000) | 1552.0 |
| Crystal size/mm^3^ | 0.18 × 0.15 × 0.11 |
| Radiation | Mo Kα (λ = 0.71073) |
| 2Θ range for data collection/° | 5.114 to 61.102 |
| Index ranges | -29 ≤ h ≤ 28, -23 ≤ k ≤ 22, -14 ≤ l ≤ 14 |
| Reflections collected | 57691 |
| Independent reflections | 10453 [R_int_ = 0.0254, R_sigma_ = 0.0180] |
| Data/restraints/parameters | 10453/0/460 |
| Goodness-of-fit on F^2^ | 1.015 |
| Final R indexes [I>=2σ (I)] | R_1_ = 0.0308, wR_2_ = 0.0787 |
| Final R indexes [all data] | R_1_ = 0.0377, wR_2_ = 0.0825 |
| Largest diff. peak/hole / e Å^-3^ | 0.57/-0.61 |


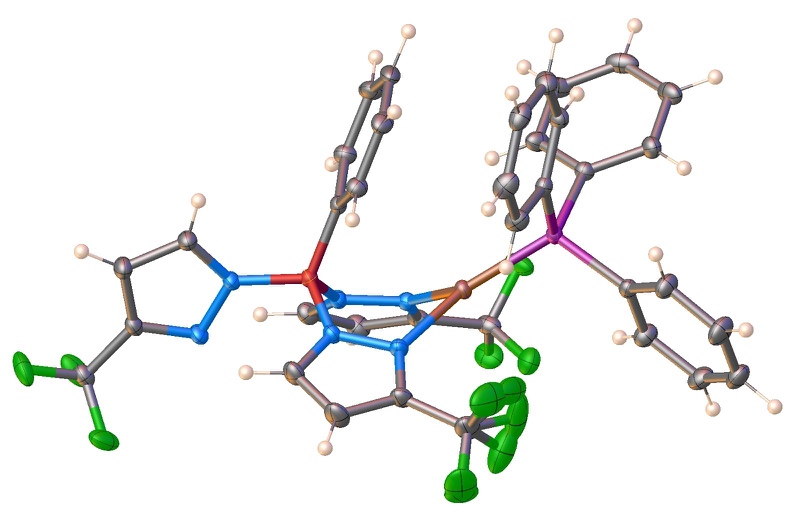


| **Table S4**. Crystal data and structure refinement for [Cu(PPh_3_){PhB(3-(CF_3_)Pz)_3_}] (**9**) | |
| --- | --- |
| Identification code | HRD244_a |
| Empirical formula | C_36_H_26_BCuF_9_N_6_P |
| Formula weight | 818.95 |
| Temperature/K | 100.00 |
| Crystal system | monoclinic |
| Space group | P2_1_/c |
| a/Å | 13.2564(12) |
| b/Å | 15.1428(14) |
| c/Å | 17.9064(17) |
| α/° | 90 |
| β/° | 99.664(4) |
| γ/° | 90 |
| Volume/Å^3^ | 3543.5(6) |
| Z | 4 |
| ρ_calc_g/cm^3^ | 1.535 |
| μ/mm^‑1^ | 0.746 |
| F(000) | 1656.0 |
| Crystal size/mm^3^ | 0.278 × 0.25 × 0.2 |
| Radiation | Mo Kα (λ = 0.71073) |
| 2Θ range for data collection/° | 5.38 to 61.008 |
| Index ranges | -18 ≤ h ≤ 18, -21 ≤ k ≤ 21, -25 ≤ l ≤ 24 |
| Reflections collected | 59606 |
| Independent reflections | 10790 [R_int_ = 0.0235, R_sigma_ = 0.0165] |
| Data/restraints/parameters | 10790/66/515 |
| Goodness-of-fit on F^2^ | 1.034 |
| Final R indexes [I>=2σ (I)] | R_1_ = 0.0286, wR_2_ = 0.0748 |
| Final R indexes [all data] | R_1_ = 0.0322, wR_2_ = 0.0769 |
| Largest diff. peak/hole / e Å^-3^ | 0.62/-0.40 |


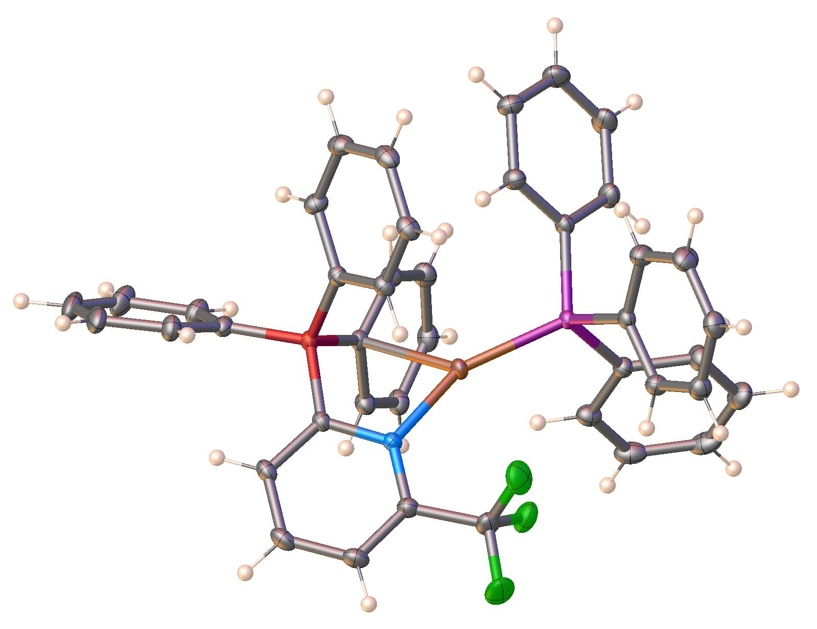


| **Table S5.** Crystal data and structure refinement for [Cu(PPh_3_){Ph_3_B(6-(CF_3_)Py)}] (**10**) | |
| --- | --- |
| Identification code | HRD166_a |
| Empirical formula | C_42_H_33_BCuF_3_NP |
| Formula weight | 714.01 |
| Temperature/K | 100.00 |
| Crystal system | triclinic |
| Space group | P-1 |
| a/Å | 9.5035(2) |
| b/Å | 12.6837(2) |
| c/Å | 15.1835(3) |
| α/° | 89.6430(10) |
| β/° | 88.6950(10) |
| γ/° | 68.3480(10) |
| Volume/Å^3^ | 1700.63(6) |
| Z | 2 |
| ρ_calc_g/cm^3^ | 1.394 |
| μ/mm^‑1^ | 0.738 |
| F(000) | 736.0 |
| Crystal size/mm^3^ | 0.35 × 0.3 × 0.26 |
| Radiation | Mo Kα (λ = 0.71073) |
| 2Θ range for data collection/° | 6.378 to 63.624 |
| Index ranges | -14 ≤ h ≤ 14, -18 ≤ k ≤ 18, -22 ≤ l ≤ 22 |
| Reflections collected | 30281 |
| Independent reflections | 11461 [R_int_ = 0.0139, R_sigma_ = 0.0166] |
| Data/restraints/parameters | 11461/0/442 |
| Goodness-of-fit on F^2^ | 1.034 |
| Final R indexes [I>=2σ (I)] | R_1_ = 0.0288, wR_2_ = 0.0773 |
| Final R indexes [all data] | R_1_ = 0.0321, wR_2_ = 0.0790 |
| Largest diff. peak/hole / e Å^-3^ | 0.64/-0.34 |


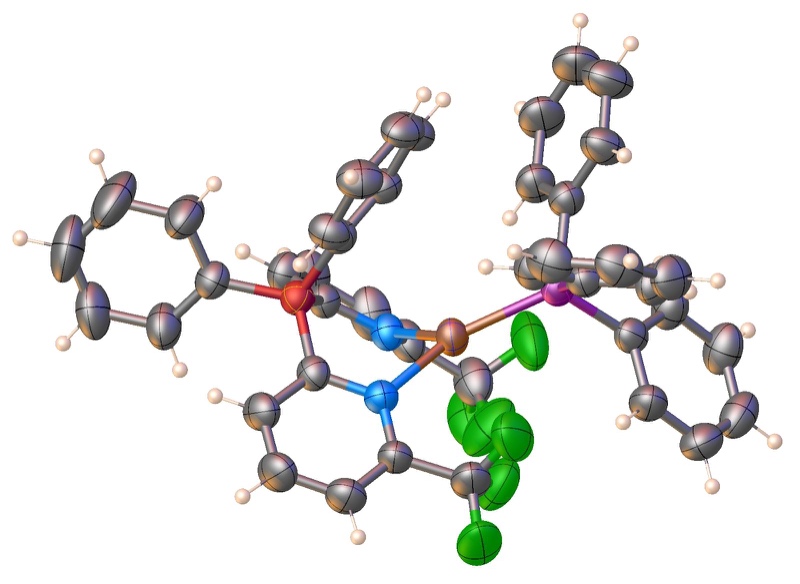


| **Table S6**. Crystal data and structure refinement for [Cu(PPh_3_){Ph_2_B(6-(CF_3_)Py)_2_}] (**11**) | |
| --- | --- |
| Identification code | HRD196_a |
| Empirical formula | C_42_H_31_BCuF_6_N_2_P |
| Formula weight | 783.01 |
| Temperature/K | 299.00 |
| Crystal system | monoclinic |
| Space group | P2_1_/n |
| a/Å | 9.7166(3) |
| b/Å | 38.2639(10) |
| c/Å | 10.1006(3) |
| α/° | 90 |
| β/° | 103.4570(10) |
| γ/° | 90 |
| Volume/Å^3^ | 3652.25(18) |
| Z | 4 |
| ρ_calc_g/cm^3^ | 1.424 |
| μ/mm^‑1^ | 0.706 |
| F(000) | 1600.0 |
| Crystal size/mm^3^ | 0.2 × 0.12 × 0.08 |
| Radiation | Mo Kα (λ = 0.71073) |
| 2Θ range for data collection/° | 5.234 to 54.96 |
| Index ranges | -12 ≤ h ≤ 12, -49 ≤ k ≤ 49, -13 ≤ l ≤ 13 |
| Reflections collected | 71921 |
| Independent reflections | 8360 [R_int_ = 0.0419, R_sigma_ = 0.0332] |
| Data/restraints/parameters | 8360/0/479 |
| Goodness-of-fit on F^2^ | 1.181 |
| Final R indexes [I>=2σ (I)] | R_1_ = 0.0601, wR_2_ = 0.1576 |
| Final R indexes [all data] | R_1_ = 0.1207, wR_2_ = 0.2223 |
| Largest diff. peak/hole / e Å^-3^ | 0.68/-0.74 |


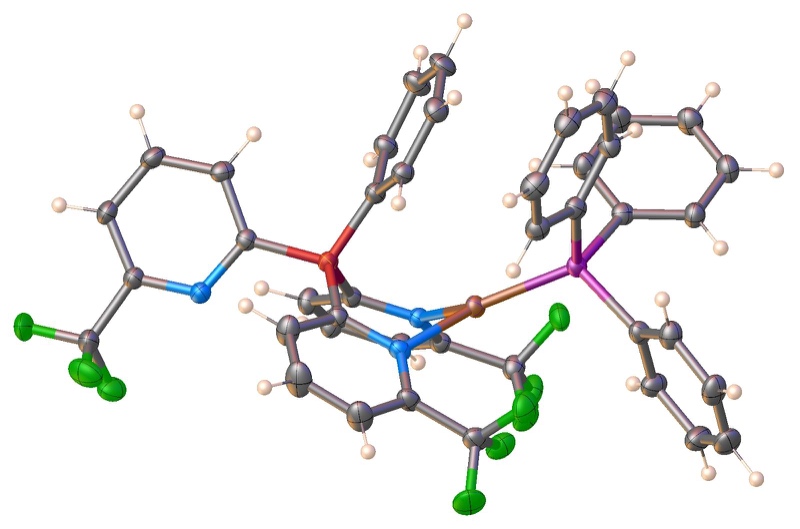


| **Table S7**. Crystal data and structure refinement for [Cu(PPh_3_){PhB(6-(CF_3_)Py)_3_}] (**12**) | |
| --- | --- |
| Identification code | HRD168_a |
| Empirical formula | C_42_H_29_BCuF_9_N_3_P |
| Formula weight | 852.00 |
| Temperature/K | 100.00 |
| Crystal system | monoclinic |
| Space group | P2_1_/c |
| a/Å | 12.7199(4) |
| b/Å | 32.4274(10) |
| c/Å | 9.6371(3) |
| α/° | 90 |
| β/° | 110.5440(10) |
| γ/° | 90 |
| Volume/Å^3^ | 3722.2(2) |
| Z | 4 |
| ρ_calc_g/cm^3^ | 1.520 |
| μ/mm^‑1^ | 0.711 |
| F(000) | 1728.0 |
| Crystal size/mm^3^ | 0.19 × 0.175 × 0.125 |
| Radiation | Mo Kα (λ = 0.71073) |
| 2Θ range for data collection/° | 4.776 to 58.262 |
| Index ranges | -17 ≤ h ≤ 17, -44 ≤ k ≤ 44, -13 ≤ l ≤ 13 |
| Reflections collected | 92863 |
| Independent reflections | 10025 [R_int_ = 0.0366, R_sigma_ = 0.0238] |
| Data/restraints/parameters | 10025/0/515 |
| Goodness-of-fit on F^2^ | 1.121 |
| Final R indexes [I>=2σ (I)] | R_1_ = 0.0549, wR_2_ = 0.1125 |
| Final R indexes [all data] | R_1_ = 0.0872, wR_2_ = 0.1428 |
| Largest diff. peak/hole / e Å^-3^ | 0.87/-0.81 |

**3. Electrochemistry**


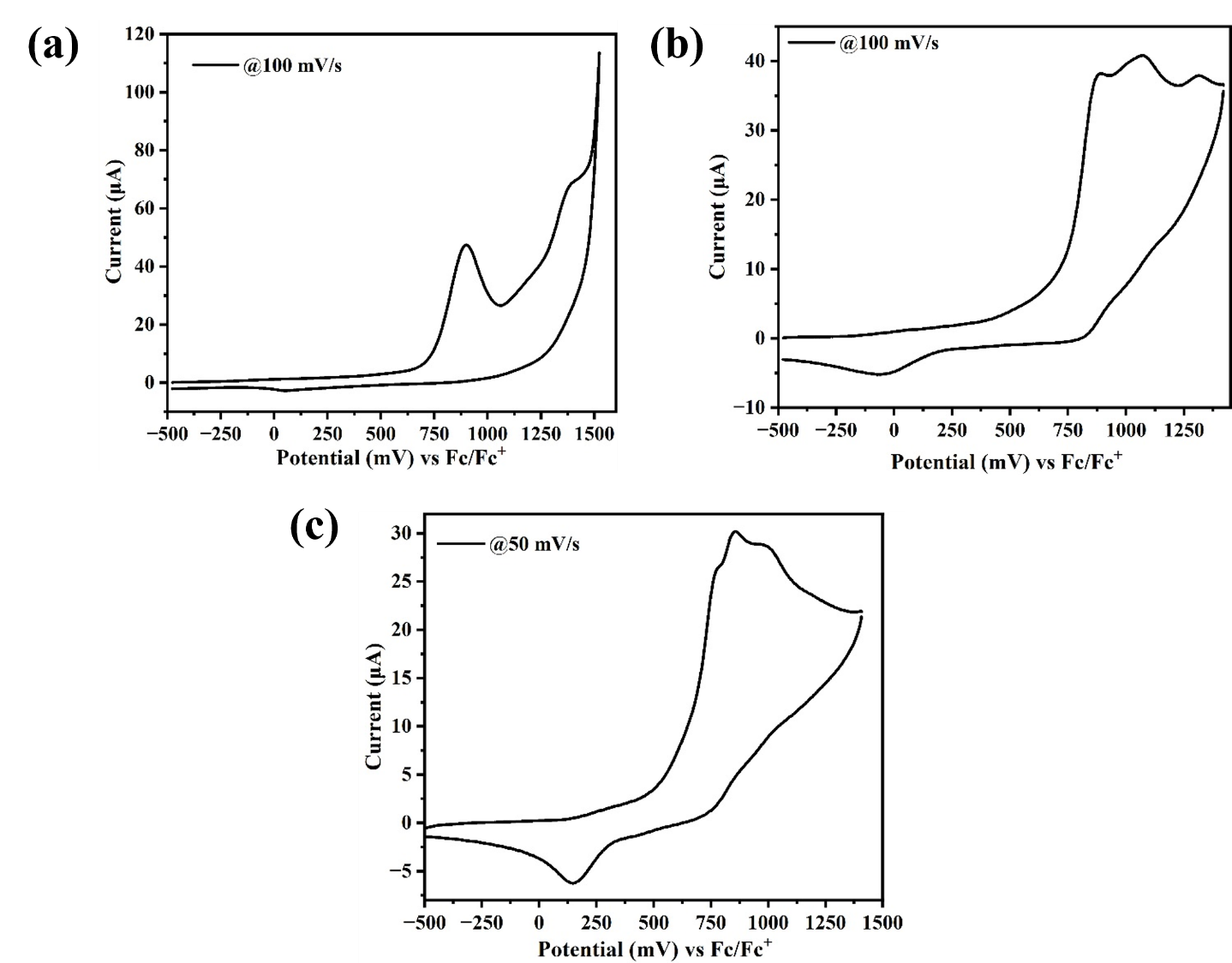


**Figure S38.** Cyclic voltammograms of 2 mM solution of complexes (a) [Cu(PPh_3_){Ph_3_B(3-(CF_3_)Pz)}] (**7**), (b) [Cu(PPh_3_){Ph_2_B(3-(CF_3_)Pz)_2_}] (**8**), and (c) [Cu(PPh_3_){PhB(3-(CF_3_)Pz)_3_}] (**9**) in 0.1 M TBAPF_6_/ACN under N_2_ atmosphere.


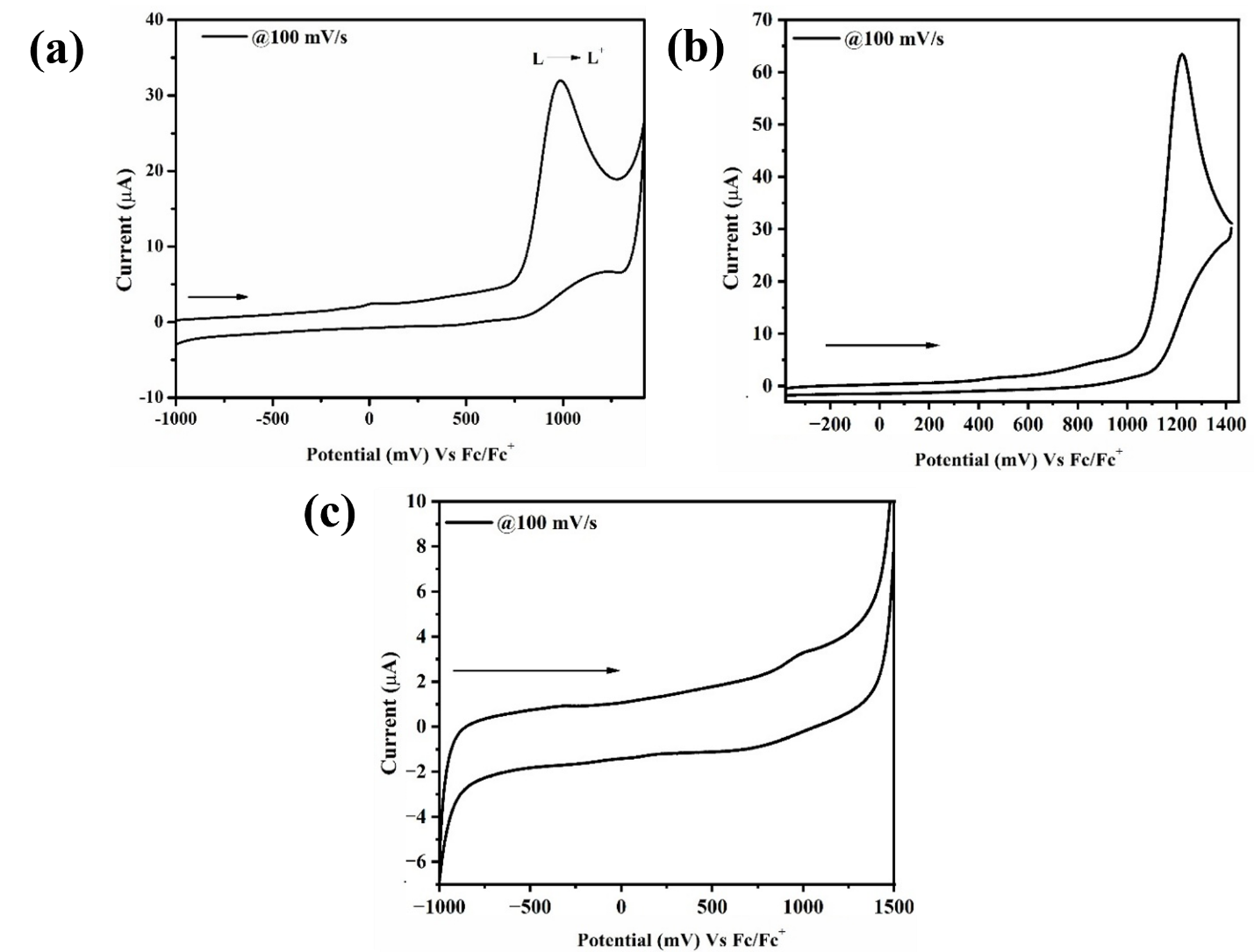


**Figure S39.** Cyclic voltammograms of 2 mM solution of complexes (a) [Ph_3_B(3-(CF_3_)Pz)]Na(THF)_2_, (b) [Ph_2_B(3-(CF_3_)Pz)_2_]Na, and (c) [PhB(3-(CF_3_)Pz)_3_]K in 0.1 M TBAPF_6_/ACN under N_2_ atmosphere.


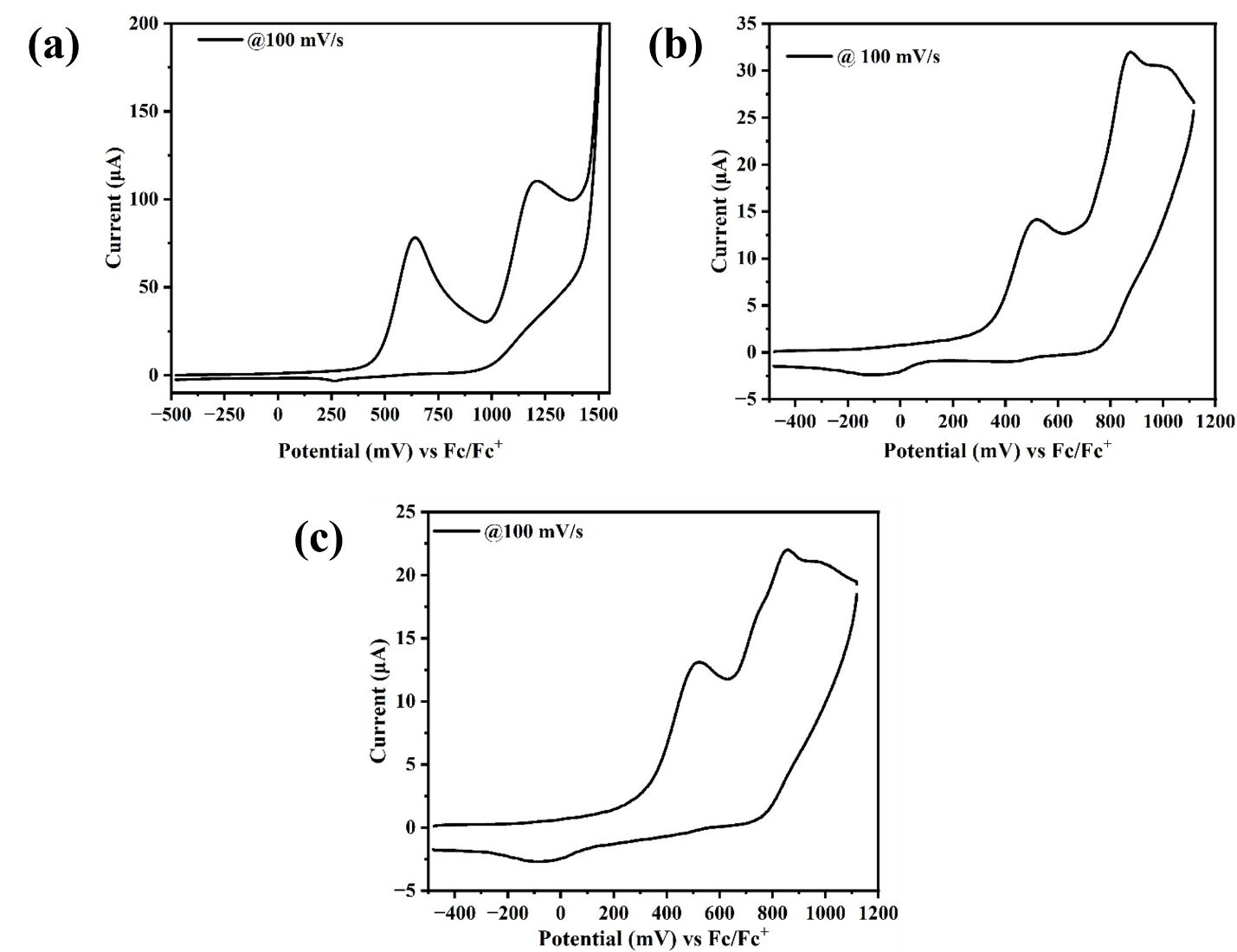


**Figure S40.** Cyclic voltammograms of 2 mM solution of complexes (a) [Cu(PPh_3_){Ph_3_B(6-(CF_3_)Py)}] (**10**), (b) [Cu(PPh_3_){Ph_2_B(6-(CF_3_)_2_Py)_2_}] (**11**), and (c) [Cu(PPh_3_){PhB(6-(CF_3_)_2_Py)_3_}] (**12**) in 0.1 M TBAPF_6_/ACN under N_2_ atmosphere.


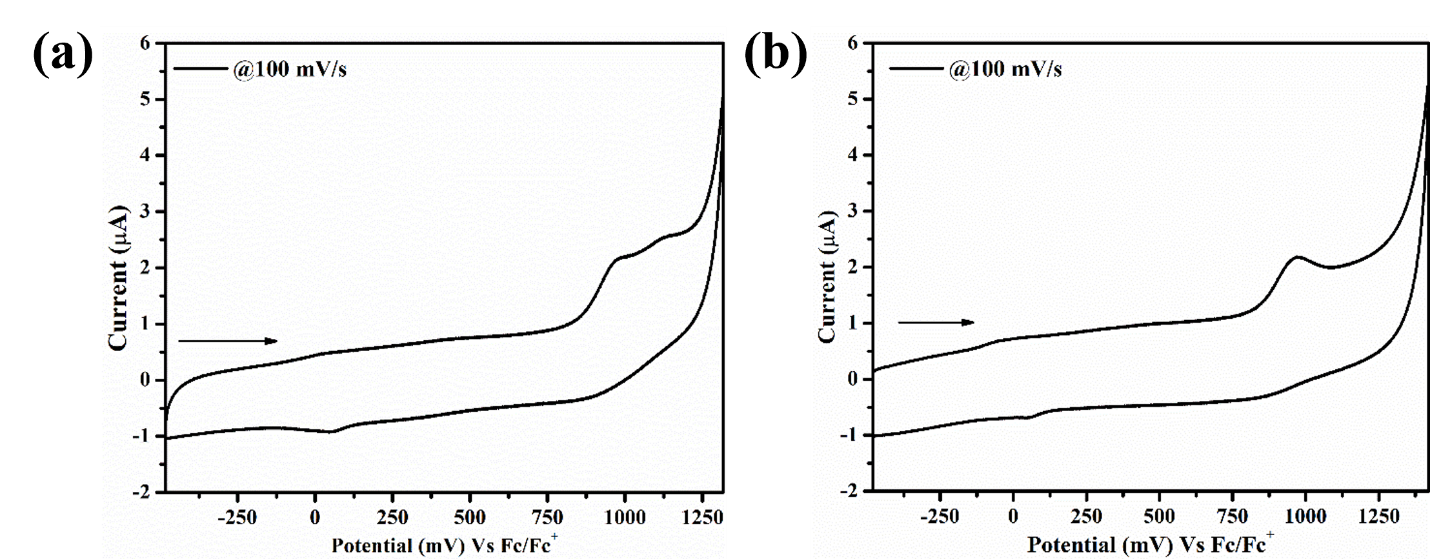


**Figure S41.** Cyclic voltammograms of 2 mM solution of complexes (a) [Ph_2_B(6-(CF_3_)_2_Py)_2_]H, and (b) [PhB(6-(CF_3_)_2_Py)_3_]H in 0.1 M TBAPF_6_/ACN under N_2_ atmosphere.

**4. References**

[1] H. V. R. Dias, W. C. Jin, H. J. Kim and H. L. Lu, *Inorg. Chem.* **1996**, *35*, 2317-2328.

[2] H. V. R. Dias, S. A. Richey, H. V. K. Diyabalanage and J. Thankamani, *J. Organomet. Chem.* **2005**, *690*, 1913-1922.

[3] H. V. R. Dias and H.-L. Lu, *Inorg. Chem.* **2000**, *39*, 2246-2248.

[4] H. V. R. Dias, G. Gioia Lobbia, G. Papini, M. Pellei and C. Santini, *Eur. J. Inorg. Chem.* **2009**, 3935-3941.
